# Supplementary material for: Distribution of aerophilous diatom communities associated with terrestrial green macroalgae in the South Shetland Islands, Maritime Antarctica
Source: PLoS One. 2019 Dec 30;14(12):e0226691. doi: 10.1371/journal.pone.0226691 (PMC6936843; doi:10.1371/journal.pone.0226691)
Supplement: Attachment — Submitted filename: PONE-D-19-16073_R1_reviewer_with notes.pdf [file pone.0226691.s002.pdf]

PLOS ONE

Distribution of aerophilous diatom communities associated with terrestrial green macroalgae in the South Shetland Islands, Maritime Antarctica

--Manuscript Draft--

**Manuscript Number:**	PONE-D-19-16073R1
**Article Type:**	Research Article
**Full Title:**	Distribution of aerophilous diatom communities associated with terrestrial green macroalgae in the South Shetland Islands, Maritime Antarctica
**Short Title:**	Diatoms associated with Antarctic green macroalgae.
**Corresponding Author:**	Filipe de Carvalho Victoria, Ph.D.<br>Universidade Federal do Pampa<br>São Gabriel, Rio Grande do Sul BRAZIL
**Keywords:**	Algae, Antarctic, Biogeography, Aerophilous diatoms.
**Abstract:**	The establishment of diatom communities depends on environmental factors such as the type of substrate and geographic conditions that influence the dispersal processes of these organisms. The main goal of this study was to evaluate the similarity between diatom communities associated with the macroalgae Prasiola crispa(Lightfoot) Kützing in relation to spatial distance from six sampled sites located in the South Shetland Islands, Maritime Antarctica. The diatom flora associated with Prasiola crispawas represented by 23 species distributed in 15 genera. Pinnularia australoschoenfelderiZidarova, Kopalová & Van de Vijver, Luticola austroatlantica Van de Vijver, Kopalová, S.A.Spaulding & EspositoEsposito, Luticolaamoenaamoena Van der Vijver, Kopalová, Zidarova & Levkov, Pinnularia austroshetlandica(Carlson) Cleve-Euler and Psammothidium papilio(D.E. Kellogg et al.) Kopalová & Zidarova were the most abundant species in our samples; together they represented 68% of the total number of individuals collected. There was great similarity in composition and abundance of the diatom communities among the sampled points, which resulted in the absence of a linear relationship pattern with distance between sampling points. We conclude that distance was not a factor of differentiation of Antarctic diatom communities associated with terrestrial green macroalgae. This suggests that Antarctic environments may have unique characteristics with homogeneous abiotic factors, at least in relation to this substrate.
**Order of Authors:**	Juliana Ferreira da Silva
	Maria Angélica Oliveira
	Raylane Ribeiro da Anunciação
	Eduardo Pereira da Silva
	Rodrigo Paidano Alves
	Adriano Luis Schünemann
	Filipe de Carvalho Victoria, Ph.D.
	Margéli Pereira de Albuquerque
	Antonio Batista Pereira
**Response to Reviewers:**	POINT-BY-POINIT RESPONSE TO REVIEWERS<br><br>REVIEWER'S COMMENTS:<br><br>Reviewer #1:<br><br>Language is generally clear, though some minor syntax and spelling editing is required throughout; I note that this journal does not offer copy editing, so this will have to be done carefully during revision. Is the citation format in the text correct for the journal? It is unusual to list both numbered references and fully named author lists together.<br>Response to reviewer: The authors followed the guidelines for manuscript formatting

Powered by Editorial Manager® and ProduXion Manager® from Aries Systems Corporation

for submission to Plos One Magazine. However, there were some formatting errors that have now been corrected in this review.

Abstract – the description of the role of distance (‘isolation by distance’) in structuring (or not) diatom community composition could be reworded a bit to make it clearer: at present the sentence structure seems to suggest initially that distance is being considered from each of the six sampling sites individually (for instance along a transect from a central sampling location at each), and rather it later becomes clear that the intention is to look at distance as a factor between the six sampling locations. Response to reviewer: Its been corrected, we rewritten the description. The modification is found in the marked copy of manuscript.

Introduction – the opening para should make it clear from the outset that the focus is on diatoms; at present the opening sentences appear to make statements applying to the Antarctic flora more generally – if this is the case then they need expanding into a separate general opening para, before subsequently focussing in on diatoms specifically. However, I think the intention is to focus on diatoms from the outset. Response to reviewer: Ok, the modification is found in the marked copy of manuscript.

Terminology in the para describing the influence of distance – it should be ‘community similarity’ rather than ‘species similarity’.
Response to reviewer: We change this, the modification is found in the marked copy of manuscript.

Within the methods section there is no reference given to how the diatoms obtained were identified? I appreciate the long list of recent species descriptions early in the Introduction, but something specific should be said in this section. Other than these comments, the approaches and analyses used are appropriate.
Response to reviewer: Ok, we add the references used in detail, the modification is found in the marked copy of manuscript.

Taxonomic authorities – I believe that journal format only requires these to be listed once, so there are many instances where repeat listings can be deleted. If acceptable to the journal, I would suggest placing all authorities within Table 2 and deleting them from the text, which will also help make the text more readable.
Response to reviewer: Ok, we change this, the modification is found in the marked copy of manuscript.

Diversity indices – I am not familiar with what 0D, 1D, 2D annotation means?
Response to reviewer: The explanation is at the bottom of page 8 in the following excerpt: “The alpha diversity was approximately 10 species for species richness and this analysis further showed that the true beta diversity of Shannon (1D) was lower than the richness based on species richness (0D) and based on the inverse concentration of Simpson (2D). This shows that differences between sampling sites occurred due to the contrast between the most abundant and the rarest species of each site (Table 4).”

Overall, while the study found differences in various indices between sites, community similarity was high across the entire study area, emphasised by the highest similarity being found between the most distant sites, and there was no overall relationship between separation distance and diversity. I wonder if this might reflect the overall physical scale of the study area, where most of the sampling sites were actually quite close to each other – in other words does this indicate the scale of the study was not large enough to identify such effects, and that ‘isolation’ was not a significant factor at this scale? Perhaps a larger geographical sampling scale would lead to identification of such a relationship, as predicted by the study’s overall hypothesis?
Response to reviewer: Ok, we added this discussion, the modification is found in the marked copy of manuscript.

Figures – the labels on the diatom photo figs are blurred in my pdf?
Response to reviewer: We do not understand this point. Our figures and the pdf generate in the submission process are in highest quality permited to the jounal guidelines.

Powered by Editorial Manager® and ProduXion Manager® from Aries Systems Corporation

Table 1 – I think I would suggest presenting this data in km rather than m
Response to reviewer: Ok, we change this, the modification is found in the marked copy of manuscript.

Reviewer #2:

Diatom identification: In the method section of the manuscript, the authors only referenced Round et al. (1990) as the source for the identification of the diatoms they observed in their samples. This is not suitable as this book by Round et al. only deals with taxonomy at the generic level and some of the genera the authors identified where not even described at the time it was published (e.g. Psammothidium, Mayamaea). It would be much better to list all the papers and flora used for identification. In addition, I found particularly surprising that you did not make use of the flora published by Zidarova et al. (2016) specifically dealing with Maritime Antarctica, including the South Shetlands Islands. In particular, the species the authors identified as Luticola aff beyensis and Luticola muticopsis morphotype 1 may be Luticola amoena and Luticola austroatlantica, respectively, as illustrated in the book by Zidarova et al. (2016). Cocconeis aff costata may also correspond to Cocconeis pinnata var. matsii as described by Al-Handal et al. (2010) from King George Island.
Response to reviewer: Ok, we correted this, the modification is found in the marked copy of manuscript.

Spelling of diatom names: I've found numerous mistakes throughout the text of the manuscript, the legend of the figures and in the tables. Please pay attention to that and correct them.
Response to reviewer: Ok, we correted this, the modification is found in the marked copy of manuscript.

Rarefaction analysis: Taxonomic richness assessed through rarefaction in fact consists in an interpolation to lower (and common to all sample) count size and it results in a loss of information (Giesecke et al. 2014). Moreover, samples standardized by size can have different degrees of completeness, depending on the species-abundance distributions of the assemblages that are compared. A better way is to compare samples of equal completeness, not equal size (Chao and Jost 2012). This can be done by using the species accumulation curve to extrapolate the total species richness of the less complete samples (Béguinot 2015). Several parameters can be used to estimate the completeness of the sampling such as the percentages of species represented by one individual (=singleton) and the sample intensity, i.e. abundance divided by richness (Lopez et al. 2012).
Response to reviewer: The authors understand the suggestion, but evaluate that would not modify the results to a significant extent. Regarding the suggestion to use species represented by only one individual in a given sample to access completeness, it cannot be applied to our data, because such species are eliminated [illegible]rom data.

Comparison of the results: the main result is that spatial distance does not appear to drive differentiation in diatom communities in this study. Maybe this result is due to the fact that the distance covered in the study is relatively small (less than 100 km). Could you compare your result with similar studies dealing with the effect of spatial distance on diatom community composition?
Response to reviewer: Ok, w[illegible]dded this discussion, the modification is found in the marked copy of manuscript.

Conclusion: I think the manuscript would really gain if you could broaden a little the scope of your conclusions with a few sentences. For example, could you discuss about any wider implications regarding the potential effect of further climate warming on the resilience of this community. I read that the penguin populations were threatened as their conditions for nesting are changing (change in local weather with unprecedented rain, prematurely snow melt creating puddles of water on the ground, etc…). How would this affect Prasiola and its associated diatom flora?
Response to reviewer: Ok, we added this discussion, the modification is found in the marked copy of manuscript.

Specific comments:
Page 1: In the title, I would use: Distribution of aerophilous diatom communities … to

Powered by Editorial Manager® and ProduXion Manager® from Aries Systems Corporation

	make it clear what kind of samples were investigated.<br>Page 1: in the address of the authors, correct spelling of Antarctic (only one "t")<br>Page 2, in abstract: rephrase such as: …such as the type of substrate…<br>Page 2: correct spelling of Luticola muticopsis (not multicopsis)<br>Page 2: add dots after initials such as: D.E. Kellogg et al.<br>Page 2:… in our samples, …<br>Page 2: …and abundance of their diatom community…<br>Page 2: keywords: replace "Diatom" by "aerophilous diatoms", delete "Taxonomy" as this paper does not deal with taxonomy<br>Page 3: rewrite the sentence such as: The larger the distance between sites, the larger are the environmental variations and, therefore, the difference in species composition tends to be also larger. The following sentence, starting by "Spatial distance is a factor that influences…" is unclear, please re-write it.<br>Page 4: quantitative analyses: give the number of valves counted to obtain the relative abundance data<br>Page 4: the statement starting with "sample sufficiency…" is unclear<br>Page 4: regarding rarefaction, please see above in general comments<br>Page 5: separate thousands using a comma such as: 10,000<br>Page 5: use lower case for "also"<br>Page 5: …(Oksanen et al. 2016) in R.<br>Page 5: correct spelling of Pseudogomphonema and carlinii<br>Page 5: Cocconeis pinnata var. matsii is also a marine species (if it's the correct identification?)<br>Page 6: correct spelling for muticopsis (several times)<br>Page 6: just give the authorities once, when a species is first mentioned. There is no need to repeat them every time thereafter.<br>Page 7: separate thousands using a comma such as: 10,000<br>Page 9: delete "1." before "Hamsher"<br>Page 9: just use Fottea for title of the journal, delete "Czech Phycological Society – Praha, Czech Republic, 2007, currens."<br>Page 9: in the reference by Kochman-Kedziora et al.: just use Fottea for title of the journal, delete "Czech Phycological Society – Praha, Czech Republic, 2007, currens."<br>Page 10: delete "3." before "Kopalova"<br>Page 11: use capital L for journal title: Limnetica<br>Page 11: delete "2." before "Van de Vijver"<br>Response to reviewer: Ok, we corrected the specific comments above.<br><br>Fig. 1: add an insert showing the whole of Antarctica to help the reader immediately visualize the location of this region. Some locations on the map and the legend are in Portuguese (e.g Rei George instead of King George; Sistema de Coordenadas, etc…). Please change all to English. Also correct spelling of British. Also give explanation about the units used on sides of the figure.<br>Response to reviewer: Ok, we correted this, the modification is found in the marked copy of manuscript.<br><br>Figs 2 and 3: indicate to what distance the scale bar corresponds (is it 5 microns?), either directly on the figures or in the legend. In the legend: add "LM photographs"; correct spelling of diatom names, use English instead of Portuguese for morphotype<br>Response to reviewer: Ok, we correted this, the modification is found in the marked copy of manuscript.<br><br>Fig. 4: add "SEM photographs", correct spelling of diatom names.<br>Response to reviewer: Ok, we correted this, the modification is found in the marked copy of manuscript.<br><br>Table 1: I would give the distances in kilometers instead of meters. If you insist in using meters, at least delete the decimals…<br>Response to reviewer: Ok, we correted this, the modification is found in the marked copy of manuscript.<br><br>Table 2: There are many mistakes in the diatom names:<br>Muticopsis instead of multicopsis<br>Cyclotella instead of Ciclotella<br>Fragilaria instead of Fragillaria

Powered by Editorial Manager® and ProduXion Manager® from Aries Systems Corporation

	Hantzschia instead of Hantzchia<br>Carlinii instead of carlini<br>Pseudogomphonema instead of Pseudoghophonema<br>Olegsakharovii instead of olegsakharoni<br>Response to reviewer: Ok, we correted this, the modification is found in the marked copy of manuscript.
**Additional Information:**	
**Question**	**Response**
**Financial Disclosure**<br><br>Enter a financial disclosure statement that describes the sources of funding for the work included in this submission. Review the submission guidelines for detailed requirements. View published research articles from *PLOS ONE* for specific examples.<br><br>This statement is required for submission and **will appear in the published article** if the submission is accepted. Please make sure it is accurate.<br><br>**Unfunded studies**<br>Enter: *The author(s) received no specific funding for this work.*<br><br>**Funded studies**<br>Enter a statement with the following details:<br>• Initials of the authors who received each award<br>• Grant numbers awarded to each author<br>• The full name of each funder<br>• URL of each funder website<br>• Did the sponsors or funders play any role in the study design, data collection and analysis, decision to publish, or preparation of the manuscript?<br>• **NO** - Include this sentence at the end of your statement: *The funders had no role in study design, data collection and analysis, decision to publish, or preparation of the manuscript.*<br>• **YES** - Specify the role(s) played.<br><br>* typeset	NO
**Competing Interests**<br><br>Use the instructions below to enter a competing interest statement for this submission. On behalf of all authors, disclose any competing interests that	NO

could be perceived to bias this work—acknowledging all financial support and any other relevant financial or non-financial competing interests.<br><br>This statement **will appear in the published article** if the submission is accepted. Please make sure it is accurate. View published research articles from *PLOS ONE* for specific examples.<br><br>**NO authors have competing interests**<br><br>Enter: *The authors have declared that no competing interests exist.*<br><br>**Authors with competing interests**<br><br>Enter competing interest details beginning with this statement:<br><br>*I have read the journal's policy and the authors of this manuscript have the following competing interests: [insert competing interests here]*<br><br>* typeset	
**Ethics Statement**<br><br>Enter an ethics statement for this submission. This statement is required if the study involved:<br><br>• Human participants<br>• Human specimens or tissue<br>• Vertebrate animals or cephalopods<br>• Vertebrate embryos or tissues<br>• Field research<br><br>Write "N/A" if the submission does not require an ethics statement.<br><br>General guidance is provided below. Consult the submission guidelines for detailed instructions. **Make sure that all information entered here is included in the Methods section of the manuscript.**	All collections were authorized by the Secretariat of the Interministerial Commission for Sea Resources (SECIRM) and endorsed by the Brazilian Government's Ministry of the Environment, under the activities regulated by the Brazilian Antarctic Program (PROANTAR). The permits can found in the submission as "Other" files.

Powered by Editorial Manager® and ProduXion Manager® from Aries Systems Corporation

**Format for specific study types**<br><br>**Human Subject Research (involving human participants and/or tissue)**<br>• Give the name of the institutional review board or ethics committee that approved the study<br>• Include the approval number and/or a statement indicating approval of this research<br>• Indicate the form of consent obtained (written/oral) or the reason that consent was not obtained (e.g. the data were analyzed anonymously)<br><br>**Animal Research (involving vertebrate animals, embryos or tissues)**<br>• Provide the name of the Institutional Animal Care and Use Committee (IACUC) or other relevant ethics board that reviewed the study protocol, and indicate whether they approved this research or granted a formal waiver of ethical approval<br>• Include an approval number if one was obtained<br>• If the study involved *non-human primates*, add *additional details* about animal welfare and steps taken to ameliorate suffering<br>• If anesthesia, euthanasia, or any kind of animal sacrifice is part of the study, include briefly which substances and/or methods were applied<br><br>**Field Research**<br><br>Include the following details if this study involves the collection of plant, animal, or other materials from a natural setting:<br>• Field permit number<br>• Name of the institution or relevant body that granted permission	
**Data Availability**<br><br>Authors are required to make all data underlying the findings described fully available, without restriction, and from the time of publication. PLOS allows rare exceptions to address legal and ethical concerns. See the PLOS Data Policy and FAQ for detailed information.	Yes - all data are fully available without restriction

A Data Availability Statement describing where the data can be found is required at submission. Your answers to this question constitute the Data Availability Statement and **will be published in the article**, if accepted.<br><br>**Important:** Stating 'data available on request from the author' is not sufficient. If your data are only available upon request, select 'No' for the first question and explain your exceptional situation in the text box.<br><br>Do the authors confirm that all data underlying the findings described in their manuscript are fully available without restriction?	
**Describe where the data may be found in full sentences. If you are copying our sample text, replace any instances of XXX with the appropriate details.**<br><br>• If the data are **held or will be held in a public repository**, include URLs, accession numbers or DOIs. If this information will only be available after acceptance, indicate this by ticking the box below. For example: *All XXX files are available from the XXX database (accession number(s) XXX, XXX.*).<br>• If the data are all contained **within the manuscript and/or Supporting Information files**, enter the following: *All relevant data are within the manuscript and its Supporting Information files.*<br>• If neither of these applies but you are able to provide **details of access elsewhere**, with or without limitations, please do so. For example:<br><br>*Data cannot be shared publicly because of [XXX]. Data are available from the XXX Institutional Data Access / Ethics Committee (contact via XXX) for researchers who meet the criteria for access to confidential data.*<br><br>*The data underlying the results presented in the study are available from (include the name of the third party*	All relevant data are within the manuscript and its Supporting Information files. The protocols used in the presente work are submitted to protocols.io platform.

and contact information or URL).<br>• This text is appropriate if the data are owned by a third party and authors do not have permission to share the data.<br><br>* typeset	
Additional data availability information:	

Manuscript
Click here to access/download;Manuscript;renamed_30d3d.docx

Distribution of aerophilous diatom communities associated with terrestrial green macroalgae in the South Shetland Islands, Maritime Antarctica

Juliana Ferreira da Silva[1]
Antarctic Vegetation Studies Center. Federal University of Pampa. Antonio Trilha Avenue, 1847, 97300-000, São Gabriel, RS, Brazil
Maria Angélica Oliveira
Laboratory of Phycology. Federal University of Santa Maria. Roraima Avenue, 1000, 97105-900, Santa Maria, RS, Brazil
Raylane Ribeiro da Anunciação
Laboratory of Phycology. Federal University of Pernambuco. Dom Manuel de Medeiros Street, 52171-900, Recife, PE, Brazil
Eduardo Pereira da Silva
Antarctic Vegetation Studies Center. Federal University of Pampa. Antonio Trilha Avenue, 1847, 97300-000, São Gabriel, RS, Brazil
Rodrigo Paidano Alves
Max Planck Institute for Chemistry. Andre Araujo Avenue, 2936, 69067-375, Manaus, AM, Brazil
Adriano Luis Schünemann
Antarctic Vegetation Studies Center. Federal University of Pampa. Antonio Trilha Avenue, 1847, 97300-000, São Gabriel, RS, Brazil
Filipe de Carvalho Victoria*
Antarctic Vegetation Studies Center. Federal University of Pampa. Antonio Trilha Avenue, 1847, 97300-000, São Gabriel, RS, Brazil, filipevictoria@unipampa.edu.br
Margéli Pereira de Albuquerque
National Institute of Antarctic Science and Tecnology – INCT/APA, Antarctic Vegetation Studies Center. Federal University of Pampa. Antonio Trilha Avenue, 1847, 97300-000, São Gabriel, RS, Brazil
and Antônio Batista Pereira
Antarctic Vegetation Studies Center. Federal University of Pampa. Antonio Trilha Avenue, 1847, 97300-000, São Gabriel, RS, Brazil

Running title: Diatoms associated with Antarctic green macroalgae.

* Corresponding Author

ABSTRACT

The establishment of diatom communities depends on environmental factors such as the type of substrate and geographic conditions that influence the dispersal processes of these organisms. The main goal of this study was to evaluate the similarity between diatom communities associated with the macroalgae *Prasiola crispa* (Lightfoot) Kützing in relation to spatial distance from six sampled sites located in the South Shetland Islands, Maritime Antarctica. The diatom flora associated with *Prasiola crispa* was represented by 23 species distributed in 15 genera. *Pinnularia australoschoenfelderi* Zidarova, Kopalová & Van de Vijver, *Luticola austroatlantica* Van de Vijver, Kopalová, S.A.Spaulding & EspositoEsposito, *Luticola amoenaamoena* Van der Vijver, Kopalová, Zidarova & Levkov, *Pinnularia austroshetlandica* (Carlson) Cleve-Euler and *Psammothidium papilio* (D.E. Kellogg et al.) Kopalová & Zidarova were the most abundant species in our samples; together they represented 68% of the total number of individuals collected. There was great similarity in composition and abundance of the diatom communities among the sampled points, which resulted in the absence of a linear relationship pattern with distance between sampling points. We conclude that distance was not a factor of differentiation of Antarctic diatom communities associated with terrestrial green macroalgae. This suggests that Antarctic environments may have unique characteristics with homogeneous abiotic factors, at least in relation to this substrate.

Keywords: Algae, Antarctic, Biogeography, Aerophilous diatomsds

INTRODUCTION

The diatom species diversity of the Antarctic is limited by extreme conditions, and studies have led to the conclusion that not all Antarctic islands share similar flora, although the overall diversity of this flora on the continent is limited compared to tropical and subtropical regions [1]. Furthermore, many of the diatom taxa recently described are endemic to Antarctica and not cosmopolitan [2,3]. Regarding the study of diatom communities, in recent years, a more refined taxonomy has revealed a large number of new species in Antarctica [3-16].

Among biological substrates where microalgal communities may grow, terrestrial macroscopic algae (though unexpected at first) may be included. The class Trebouxiophyceae includes a group of morphologically heterogeneous eukaryotic green algae that occur mainly in soil and continental waters. The genus *Prasiola* Meneghini belongs to this class and includes marine, terrestrial and continental species. *Prasiola crispa* (Lightfoot) Kützing is a terrestrial species of Antarctica, which usually grows on moist soils that are fertilized by bird guano, being more abundant inside and around penguin colonies. These macroalga tolerates repeated cycles of freezing and thawing during the year, as well as high levels of UV radiation during the summer [17]. At the micro-scale level, the substrate of periphytic communities may provide more than an inert surface, since its physical and chemical characteristics can influence the composition of the community and algal biomass [18-20].

Studies that address distribution patterns often document a decrease in species similarity as spatial distance increases between communities [21-23]. This reduction of similarity is attributed to some major factors such as environmental conditions and species dispersal processes. Spatial distance is a factor that influe[illegible], the larger the distance between sites, the larger are the environmental variations and, therefore, the th[illegible]fference between species tends to be also larger. Spatial distance is a factor that influences the displacement of species across smaller distances, once that this factor increases the chances of survival, [illegible]erent from migration over greater distances [21]. The main goal of this study was to evaluate the similarity between diatom communities associated with [illegible]*rispa* spatially distributed in six sites located in the South Shetland Islands, Antarctica. The proposed hypothesis is that the larger the spatial distance between sampling points, the smaller the similarity of diatom communities associated with *P. crispa*.

MATERIAL AND METHODS

During the Brazilian Antarctic Expeditions XXXIII (2014-2015) and XXXIV (2015-2016) (Fig. 1), samples of *P. crispa* where obtained from Ardley Island, Halfmoon Island and King George Island (Copacabana, Punta Plaza, Steinhoose and Voureal), in the Antarctica Maritime. All collections were authorized by the Secretariat of the Interministerial Commission for Sea Resources (SECIRM) and endorsed by the Brazilian Government's Ministry of the Environment, under the activities regulated by the Brazilian Antarctic Program (PROANTAR).

Table 1– Sample site coordinates.

Sample Site	Latitude	Longitude
Ardley Island	62°12'40.40"S	058°55'38.70"W
Halfmonn Island	62°35'41.5"S	059°55'10.1"W
King George Island: Copacabana	62°10'38.4"S	058°26'57.3"W
King George Island: Punta Plaza	62°5'14.69"S	058°23'35.21"W
King George Island: Steinhoose	62°04'18.9"S	058°22'28.2"W
King George Island: Voureal	62°10'2.6"S	058°17'52.0"W

The samples of *P. crispa* were stored in sealed plastic bags and frozen for further analysis in the Laboratory of Phycology of the Federal University of Santa Maria (UFSM), Rio Grande do Sul, Brazil. To prepare the material, cell contents and organic matter of the samples were removed by oxidation with hydrogen peroxide (H_2O_2) and potassium permanganate ($KMnO_4$) [24]. After washing with distilled water, permanent slides were mounted using Naphrax (refractive index of 1.74, Brunel Microscopes Ltd, Chippenham, Wiltshire, United Kingdom). Qualitative and quantitative analyses were carried out under a Leica DM750 optical microscope. Quantitative analyses were performed with approximately 80 valves [illegible] each sample, because the abundance is very small in the substrate. Thus, it was necessary to prepare a lot of permanent slides to make observations under this criteria. Taxa were identified following Round et al. [25]. A specific bibliography for identifying diatoms from polar environmental was used [1, 8-11; 14-15, 26-43].

For data analysis, the spatial distance in metres between each pair of sampling sites was calculated based on the registered geographical points. Thus, a geographic distance matrix (Table 2) was constructed for further comparison using the matrices from biological similarity of relative abundance (abundance of individuals by taxa) (Table 3) and species composition (presence and absence of taxa in points).

The estimation of species richness was based on a simple arithmetic of the estimators, without consideration of standard deviations [44]. The samples sufficiency was calculated based on the percentage of values observed in relation to the estimated values. In order to compare the species richness among the sample points, individual

rarefaction curves were constructed, and compared in the lowest abundance value among them (N = 41), in order to correct for differences in size and sample effort. For this purpose, the abundance data of each species were used for each sampling point. The EstimateS program (Department of Ecology & Evolutionary Biology, University of Connecticut, Storrs, USA) was used for analyses, with 100 randomisations of the data [45]. Rank-abundance diagrams were constructed to describe and compare the distribution of abundance and dominance of species at each sampling site using relative abundance data [46]. To describe these distributions numerically, the Pielou equitability index was adopted, based on the Shannon diversity index.

Table 2. Distance table in kilometers between sampling points where *Prasiola crispa* was collected in Antarctica.

Sample sites	Ardley	Copacabana	Halfmoon	Punta Plaza	Steinhouse	Voureal
Ardley	0					
Copacabana	25.17	0				
Halfmoon	66.76	89.14	0			
Punta Plaza	31.07	10.43	97.19	0		
Steinhouse	32.74	12.37	99.00	1.98	0	
Voureal	33.13	7.96	96.46	10.20	10.55	0

Table 3. List of all observed species with their acronyms and abundance in the samples from: Ard= Ardley; Cop= Copacabana; Hal=Half Moon; PuP= Punta Plaza; Ste= Steinhoose; Vou= Voureal. Species

Species	Acronyms	Sample points: A rd	C o	H al	P u	St e	V o
Luticola amoena Van der Vijver, Kopalová, Zidarova & Levkov	A	0	0	0	0	5.	0
Luticola austroatlantica Van de Vijver, Kopalová, S.A. Spaulding & Esposito	B	0	0	0	0	2.	0
Luticola muticopsis (Van Heurck) D.G. Mann	C	0	0	0	0	1.	1.
Achnanthes sp.	D	0	0	0	2.	0	0
Achnanthidium aff *indistinctum* Van de Vijver & Kopalová	E	0	1.	0	0	1	0
Cyclotella meneghiniana Kützing 18[illegible]	F	0	1.	0	0	0	0
Cocconeis pinnata var. *matsii* Al-Handal, Riaux-Gobin & Wulff	G	2.	4	1	1	7.	1
Eunotia aff *pseudopaludosa* Van de Vijver, de Haan & Lange-Bertalot	H	0	0	3	2	2	2.
Fragilaria cf *parva* Tuji & D.M. Williams	I	2.	4.	1.	3.	0.	0
Hantzschia amphioxys f. *muelleri* Ts. Ko-Bay[illegible]	J	7.	4.	0	0	0	0
Pteroncola carlinii Almandoz & Ferrario 2014	K	0	0	0	8.	7.	0
Mayamaea cf *atomus* (Kützing) Lange-Bertalot 1997	L	0	0	4.	0	0	0
Navicula aff *perminuta* Ǿstrup 1913	M	0	2.	0	0	0	0
Pinnularia aff *microstauron* (Ehrenberg) Cleve 1891	N	0	3.	3.	0	0	0
Pinnularia australoschoenfelderi Zidarova, Kopalová & Van de Vijver	O	5	0	1	0	0	5
Pinnularia austroshetlandica (Carlson) Cleve-Euler	P	2	9.	0	4.	6.	1
Pinnularia borealis Ehrenberg sensu lato	Q	1	0	3.	9.	1	0
Psammonthidium rostrogermainii Van de Vijver, Kopalová & Zidarova	R	0	2.	0	0	6.	0

Psammothidium germainii (Manguin) Sabbe	S	0	0	7.	0	3.	0
Psammothidium papilio (D.E. Kellogg et al.) Kopalová & Zidarova	T	0	1	2.	7.	8.	9.
Pseudogomphonema kamtschaticum (Grunow) L.K.Medlin, 1986	U	0	6.	4.	0	0	2.
Luticola olegsakharovii Zidarova, Levkov & Van de Vijver	V	0	0	8.	2	0	0
Thalassiosira gracilis var.expecta G. Fryxell & Hasle 1979	X	0	7.	7.	0	0	0

The diversity was partitioned into the alpha, beta and gamma components using Hill's numbers [47,48] with the “entropart” package [49] in the R program (R Foundation for Statistical Computing, Vienna, Austria) (R Core Team 2017), with the approach of multiplicative partitioning of diversity.

Jaccard and Bray-Curtis dissimilarity coefficients were used to calculate dissimilarity between pairs of sampling points based on incidence and abundance data. An array of geographical (Euclidean) distances between points was derived from the longitude and latitude coordinates. Subsequently, the Jaccard and Bray-Curtis distance matrices were regressed with the geographic distance matrix using linear regressions [50].

The standard coefficients of these regression models were used as measures of the decay rate when comparing similarity as a function of geographical distance between points [22]. In addition, biotic distance data were correlated with the geographic distance matrix in order to corroborate the relationship probability values based on 100 permutations using the Mantel test [23]. Statistical analyses were performed in the R program using the "vegan" [51] and "betapart" [52] packages, and visualised through dendrograms also a using the "vegan" package [51] in R envi-ronment.

In order to verify which component of the beta diversity predominantly contributed to the dissimilarity between sampling points, species presence/absence dissimilarity was decomposed into substitution (turnover) and nestedness [52] components, while abundance-based dissimilarity was decomposed into the components of abundance balanced variation and abundance gradients [53]. For this, Jaccard and Bray-Curtis dissimilarity coefficients were used in the "betapart" package [54] implemented in the R program. The matrices of beta diversity components based on presence/absence and abundance were also associated to the geographic distance matrix, as previously described, in order to verify which dissimilarity component was related to the spatial configuration of the sampling sites.

The protocols used for the present study can be accessedacess through protocol.io (dx.doi.org/10.17504/protocols.io.6tcheiw).

RESULTS AND DISCUSSION

The diatom flora associated with *Prasiola crispa* at the six sampling sites were represented by 23 species (Fig. 2-4) distributed in 15 genera. Two species are typical of marine environments: *Pseudogomphonemam kamtschaticum* and *Cocconeis pinnata* var. *matsii*[illegible]*matsi*[illegible]*eroncola carlinii*. The presence of these species in these terrestrial sites is probably related to their proximity to the sea.

Comparing the rarefied richness of the islands, in order to correct for sample size differences between sites, We observed that Steinhoose and Halfmoon had higher species richness, even when comparing the same number of individuals (total abundance = 41 in both sites) (Fig. 5). Intermediate richness was observed in Copacabana and Punta Plaza, and even though Voureal had the largest number of individuals, this site had similar rarefied richness to that of Ardley, which had the lowest number of species and individuals (Fig. 5).

The most abundant species were *Pinnularia australoschoenfelderi* (20.0%), *Luticola austroatlantica* (15.5%), *Luticola amoenaamo*[illegible] (15.3%), *Pinnularia austroshetlandica* (9.2%) and *Psammothidium papilio* (8.0%), which altogether represented 68% of the total number of individuals collected. There was a difference in the most abundant species between each sampling site (Fig. 6). The dominance pattern in Steinhoose and Punta Plaza, the two areas closest to each other in this study, was similar, with high abundance of *L. austroatlantica* and *Pinnularia borealis*. These two areas also had the highest Pielou equitability values (Punta Plaza: Pielou = 0.91, Steinhoose: Pielou = 0.89). However, *L. austroatlantica* was also the most abundant at the other extreme of the distance gradient in Halfmoon (Pielou = 0.86). *P. australoschoenfelderi* dominated in Ardley (Pielou = 0.73) and Voureal (Pielou = 0.71), while *L. amoenaamo*[illegible] dominated in Copacabana (Pielou = 0.76). This species was also among the three most abundant species in Halfmoon, Punta Plaza and Voureal.

The diversity partition demonstrated that true diversity based on species richness (greater weight on rare species), Shannon's entropy (similar weight on common and rare species), and Simpson index (greater weight on dominant species) showed similar response patterns, with higher values in Steinhoose (Table 4). The alpha diversity was approximately ten species for species richness; this analysis further showed that the true beta diversity of Shannon (1D) was lower than the richness based on species richness (0D) and based on the inverse concentration of Simpson (2D). This shows that differences between sampling sites occurred due to the contrast between the most abundant and the rarest species at each site (Table 3). The beta diversity values for all orders were close to 2, indicating that there are at least two large groups of species with high substitutions.

Table 4. Multiplicative partitioning of the diatom species diversity associated with *Prasiola crispa* for the sampled sites. Ard = Ardley; Half = Halfmoon; Cop = Copacabana; Pupl = Punta Plaza; Stei = Steinhoose; Vou = Voureal; true beta diversity of Shannon (^{1}D); species richness (^{0}D); concentration of Simpson (^{2}D).

	Islands						Components of diversity		
	Ard	Half	Cop	PuPl	Stei	Vou	Α	β	γ
^0D	6	12	12	9	13	7	10	2.3	23
^1D	3.73	8.45	6.68	7.31	9.75	3.95	6.38	1.92	12.26
^2D	2.91	6.17	4.22	6.32	7.92	2.88	4.44	2.01	8.92

Total dissimilarity based on the presence/absence and abundance of species between the islands was 80.7% and 80.5%, respectively. Similarly, analyses of parity-by-pair dissimilarity (both presence/absence and abundance based) showed similar patterns of response, but no formation of distinct groups (Fig. 7). The high similarity between the nearest sites (Steinhoose and Punta Plaza) with the most distant site (Halfmoon), for presence/absence as well as for number of individuals, should be emphasized. Copacabana had the highest average dissimilarity with the other sampling sites based on species composition (Fig. 7A), while Ardley had the greatest dissimilarity based on abundance of individuals (Fig. 7B).

The decomposition of beta diversity based on the presence/absence of species showed that the species substitution component was the most important relative to dissimilarity among the sampling sites (73.1%). The relative importance of the nesting component was only 7.6%, corroborating the beta diversity values slightly higher than two shown by the partitioning analysis of diversity in the alpha, beta and gamma components. These results were strongly influenced by the observed species richness in each sampling site, as well as the high number of exclusive species (26.1% of the total species). In addition, only 26.1% of the species occurred in more than 50% of the sites.

Community similarity between sampling sites was not related to the geographical distance between them, based on incidence ($t = -0.509$, $p = 0.619$) and abundance ($t = -1.041$, $p = 0.3167$) (Fig. 8A and D). Species substitution components ($t = 0.610$, $p = 0.553$) and nesting ($t = -0.403$, $p = 0.693$) of incidence-based beta diversity were also not related to geographical distance (Fig. 8B and C). Similarly, beta diversity components based on the abundance from balanced variation of abundance ($t = 0.180$, $p = 0.860$) and abundance gradients ($t = 0.654$, $p = 0.525$) were not related to the geographical distance between sampling sites (Fig. 8E and F). The Mantel tests, based on

10000 permutations, corroborated the probability values for this lack of relationship between the similarity matrix and dissimilarity components with the between-sites geographical distance. The lack of a positive or negative similarity relationship with geographical distance occurred due to the high similarity in composition and abundance among the most distant sampling points. In other words, even very close or very distant sites have high similarity in diatom composition and abundance, which resulted in the absence of a linear relationship pattern with distance.

Prasiola crispa is found in drainage lines in Antarctica, and because it is ornithocoprophilous, presents considerable biomass mainly around penguin colonies [55]. Thus, this makes nutrient availability for both the green algae and the surrounding diatom community higher, when compared to e.g. epilithic habitats. The lack of differentiation between the communities with geographic distance could also be attributed to the lower climatic variation in Antarctica compared to tropical and subtropical regions.. In a previous study of three marine red macroalgae in Newfoundland Bay, Antarctica [56], the biophysical-chemical characteristics of each sampling site affected the diatom communities more markedly than the different species of host macroalgae or even depth of sampling. Moreover, the diversity of the epiphytic community of diatoms associated with red macroalgae collected near the sea ice of Cape Evans, Antarctica, showed that species diversity decreased as the depth below the sea ice increased, whereby the dominant taxa had also changed in relation to depth [59-61].

Our study showed that even with marked distance between sampling points, there was high similarity in the composition and abundance of the diatom communities, which resulted in the absence of a linear relationship pattern with distance. However, it was possible to demonstrate that regarding dominant species, the closest sampling sites were more similar. Thus, the inexpressive differentiation of the communities showed that *P. crispa* as a substrate seems to be an important factor for the selection of the existing epiphytic community.

There have been an increasing number of studies reporting changes in the community of Antarctic organisms due to global warming. Some penguin communities are experiencing population declines as a result of rising temperatures [57], and, moss communities are increasing on King George Island, colonizing fields in uncovered areas due to shrinking glaciers [58]. Decreases in microbial diversity patterns has also been reported in regions of Patagonian and other Antarctic lakes, mainly found to decrease in diatom diversity [62], as well with evidence for the decline of phytoplankton [63], and bacterioplankton [64]. Such aspects lead us to reflect on how much the diatom communities, as well as the substrate of the present study (*P. crispa*) can be affected, since *P. crispa* grows in humid places with high nutrient input. If we eliminate these factors, we would also affect the existence of this macroalgae species and, consequently, that of the associated aerophytic diatom community. Although we have not observed any

exclusive communities present in this substrate, we believe that by affecting the stability of *P. crispa* in terrestrial wetlands, we would also affect the dispersal of associated diatoms.

ACKNOWLEDGMENT

We acknowledge the Coordination of Improvement of Higher-Level Personnel (CAPES), for the scholarship granted to the first author. The authors would also like to thank the brazilian Ministry of the Environment and the Inter-Ministerial Secretariat for Sea Resources (SECIRM) for the authorizations granted for field collection activities under the Brazilian Antarctic Program.

AUTHOR CONTRIBUTIONS

Conceptualization: Juliana Ferreira da Silva, Maria Angélica Oliveira, Antonio Batista Pereira

Data curation: Juliana Ferreira da Silva, Filipe de Carvalho Victoria, Margéli Pereira de Albuquerque, Eduardo Pereira da Silva, Raylane Ribeiro da Anunciação

Formal analysis: Juliana Ferreira da Silva, Eduardo Pereira da Silva, Raylane Ribeiro da Anunciação, Rodrigo Paidano Alves, Maria Angélica Oliveira

Funding acquisition: Antonio Batista Pereira

Methodology: Juliana Ferreria da Silva, Adriano Luis Schunemann, Maria Angélica Oliveira

Resources: Juliana Ferreria da Silva, Adriano Luis Schunemann

Visualization: Juliana Ferreira da Silva, Adriano Luis Schunemann

Writing – original draft: Juliana Ferreira da Silva, Margéli Pereira de Albuquerque, Rodrigo Paidano Alves, Filipe de Carvalho Victoria, Maria Angélica Oliveira.

REFERENCES

1. Hamsher S, Kopalova K, Kociolek JP, Zidarova R and Van De Vijver B. The genus *Nitzschia* on the South Shetland Islands and James Ross Island. Fottea. 2016; 16(1): 79-102.

2. Van De Vijver B, Gremmen NJ and Beyens L. The genus *Stauroneis* (*Bacillariophyceae*) in the Antarctic region. J Biogeogr. 2005; 32(10): 1791-1798.

3. Kopalova K, Ochyra R, Nedbalova L and Van De Vijver B. Moss-inhabiting diatoms from two contrasting Maritime Antarctic islands. Plant Ecol Evol. 2014; 147(1): 67–84.

4. Kochman-Kedziora N, Noga T, Zidarova R, Kopalová K and Van DE Vijver B. *Humidophila komarekiana* sp. nov.(*Bacillariophyta*), a new limnoterrestrial diatom species from King George Island (Maritime Antarctica). Phytotaxa. 2016; 272(3): 184-190.

5. Kochman-Kedziora N, Noga T, Van De Vijver B and Stanek-Tarkowska J. A new *Muelleria* species (*Bacillariophyta*) from the Maritime Antarctic Region. Fottea. 2017; 17(2): 264-268.

6. Kochman-Kedziora N, Pinseel E, Rybak M, Noga T, Olech M And Van De Vijver B. *Pinnularia subcatenaborealis* sp. nov.(*Bacillariophyta*) a new chain-forming diatom species from King George Island (Maritime Antarctica). Phytotaxa. 2018; 364(3): 259-266.

7. Kociolek JP, Kopalová K, Hamsher SE, Kohler TJ, Van De Vijver B, Convey P and Mcknight DM. Freshwater diatom biogeography and the genus *Luticola*: an extreme case of endemism in Antarctica. Polar Biol. 2017; 40(6): 1185–1196.

8. Van De Vijver B, Sterken M, Vyverman W, Mataloni G, Nedbalová L, Kopalová K, Elster J, Verleyen E and Sabbe K. Four new non-marine diatom taxa from the Subantarctic and Antarctic regions. Diatom Res. 2010; 25(2): 431-443.

9. Van De Vijver B and Zidarova R. Five new taxa in the genus *Pinnularia* sectio *Distantes* (*Bacillariophyta*) from Livingston Island (South Shetland Islands). Phytotaxa. 2011; 24: 39–50.

10. Van De Vijver B, Zidarova R and De Haan M. Four new *Luticola* taxa (*Bacillariophyta*) from the South Shetland Islands and James Ross Island (Maritime Antarctic Region). Nova Hedwigia. 2011; 92(1): 137–158.

11. Van De Vijver B, Wetzel C, Kopalová K, Zidarova R and Ector L. Analysis of the type material of *Achnanthidium lanceolatum* Brébisson ex Kützing (*Bacillariophyta*) with the description of two new *Planothidium* species from the Antarctic Region. Fottea. 2013; 13(2): 105-117.

12. Van De Vijver B, Kopalová K, Zidarova R and Kociolek JP. Two new *Gomphonema* species (B*acillariophyta*) from the Maritime Antarctic Region. Phytotaxa. 2016; 269(3): 209-220.

13. Van De Vijver B, Lange-Bertalot H, Wetzel CE and Ector L. *Michelcostea*, a new diatom genus (*Bacillariophyta*) from the sub-Antarctic Region. Nova Hedwigia. 2018; 125-136.

14. Zidarova R. Revision of the genus *Hantzschia* (Bacillariophyceae) on Livingston Island (South Shetland Islands, Southern Atlantic Ocean). Plant Ecol Evol. 2010; 143(3): 318–333. https://doi.org/10.5091/plecevo.2010.402

15. Zidarova R, Kopalová K and Van De Vijver B. The genus *Pinnularia* (*Bacillariophyta*) excluding the section *Distantes* on Livingston Island (South Shetland Islands) with the description of twelve new taxa. Phytotaxa. 2012; 44(1):11-37.

16. Zidarova R, Kopalová K, Van de Vijver B.. Diatoms from the Antarctic region, Maritime Antarctica. Schmitten-Oberreifenberg: Koeltz Botanical Books, 2016.

17. Guiry MD and Guiry GM. AlgaeBase. 2016. Available at: http://www.algaebase.org (last accessed 16 May 2016).

18. Murdock JN and Dodds WK. Linking benthic algal biomass to stream substratum topography 1. J Phycol. 2007; 43(3): 449-460.

19. Bergey EA. Does rock chemistry affect periphyton accrual in streams?. Hydrobiologia. 2008; 614(1): 141-150.

20. Santos TR and Ferragut C. The successional phases of a periphytic algal community in a shallow tropical reservoir during the dry and rainy seasons. Limnetica. 2013; 32(2): 337-352.

21. Nekola JC and White PS. The distance decay of similarity in biogeography and ecology. J Biogeogr. 1999; 26: 867-878.

22. Wetzel CE, Bicudo DDC, Ector L, Lobo EA, Soininen J, Landeiro VL and Bini LM. Distance decay of similarity in neotropical diatom communities. PLoS One. 2012; 7(9): e45071.

23. Goldenberg Vilar A, Van Dam H, Van Loon EE, Vonk JA, Van Der Geest HG and Admiraal W. Eutrophication decreases distance decay of similarity in diatom communities. Freshw Biol. 2014; 59(7): 1522-1531.

24. Kelly MG, Cazaubon A, Coring E, Dell'Uomo A, Ector L, Goldsmith B, Guasch H, Hurlimann J, Jarlman A, Kawecka B, Kwandrans J, Laugaste R, Lindstrom E, A, Leitao M, Marvan P, Padisaka J, Pipp E, Prygiel J, Rott E, Sabater S, Van Dam H, Vizinet J, Recommendations for the routine sampling of diatoms for water quality assessments in Europe. J Appl Phycol. 1998; 10(2): 215–224.

25. Round FE, Crawford RM and Mann DG. Diatoms: Biology and Morphology of the Genera, Cambridge: Cambridge University Press, 747 p. 1990.

26. Al-Handal AY, Riaux-Gobin C, and Wulff A. Cocconeis pottercovei sp. nov. and Cocconeis pinnata var. matsii var. nov., Two new marine diatom taxa from King George Island, Antarctica. Diatom Res. 2010; 25(1), 1-11.

27. Kopalová K, Nedbalova L, De Haan M, Van de Vijver B. Description of five new species of the diatom genus *Luticola* (Bacillariophyta, Diadesmidaceae) found in lakes of James Ross Island (Maritime Antarctic Region). Phyto-taxa. 2011; 27 (1): 44-60. https://doi.org/10.11646/phytotaxa.27.1.5

28. Bonaventura SM, Vinocur A, Allende L, Pizarro H. Algal structure of the littoral epilithon in lentic water bodies at Hope Bay, Antarctic Peninsula. Polar Biol. 2006; 29(8): 668-680. https://doi.org/10.1007/s00300-005-0104-3

29. Esposito RMM, Spaulding DM, McKnight DM, Van de Vijver B, Kopalová K, Lubinski D, Hall B, Whittaker T. Inland diatoms from the McMurdo Dry Valleys and James Ross Island, Antarctica. Botany. 2008; 86: 1378-1392. https://doi.org/10.1139/B08-100

30. Kawecka B, Olech M, Nowogrodzka-Zagórska M, Wojtuń B. Diatom communities in small water bodies at H. Arctowski Polish Antarctic Station (King George Island, South Shetland Islands, Antarctica). Polar Biolbiology. 1998; 19(3): 183-192. https://doi.org/10.1007/s003000050233

31. Lobo EA, Callegaro VLM, Oliveira MA. Diatoms from two lakes of Peninsula Keller, King George Island, Antartic. Revista de Pesquisa Série Botânica. 1998; 10(1): 3-25.

32. Luo W, Li H, Gao S, Yu Y, Lin L, Zeng Y. Molecular diversity of microbial eukaryotes in sea water from Fildes Peninsula, King George Island, Antarctica. Polar Biol. 2016; 39(4): 605-616. https://doi.org/10.1007/s00300-015-1815-8

33. Mataloni G, Vinocur A, Pinto PT. Abiotic characterization and epilithic communities of a naturally enriched stream at Cierva Point, Antarctic Peninsula. Antarct Sci. 2005; 17(2): 163-170. https://doi.org/10.1017/S0954102005002579

34. Pizarro H, Vinocur A. Epilithic biomass in an outflow stream at Potter Peninsula, King George Island, Antarctica. Polar Biol, 2000; 23: 851-857. https://doi.org/10.1007/s003000000164

35. Procopiak LK, Fernandes LF. Valve morphology of the benthic diatom *Fallacia marnieri* (Manguin) Witkowski (Sellaphoraceae-Bacillariophyta). Brazil J Biol. 2003; 63(1): 113-119. https://doi.org/10.1590/S1519-69842003000100015

36. Van de Vijver B, Gremmen N, Smith V. Diatom communities from the sub-Antarctic Prince Edward Islands: diversity and distribution patterns. Polar Biol. 2008; 31(7): 795-808. https://doi.org/10.1007/s00300-008-0418-z

37. Van de Vijver B, Mataloni G. New and interesting species in the genus *Luticola* DG Mann (Bacillariophyta) from Deception Island (South Shetland Islands). Phycologia. 2008; 47(5): 451-467. https://doi.org/10.2216/07-67.1

38. Van de Vijver B, Agius JT, Gibson JA, Quesada A. An unusual spine-bearing *Pinnularia* species from the Antarctic Livingston Island (South Shetland Islands). Diatom Res. 2009; 24(2): 431-441. https://doi.org/10.1080/0269249X.2009.9705812

39. Van de Vijver B, Beyens L. Freshwater diatom communities of the Strømness Bay area, South Georgia. Antarct Sci. 1996; 8(4): 359-368. https://doi.org/10.1017/S0954102096000533

40. Van de Vijver B, Kopalová K. Four *Achnanthidium* species (Bacillariophyta) formerly identified as *Achnanthidium minutissimum* from the Antarctic Region. Eur J Taxon. 2014; 79: 1-19. https://doi.org/10.5852/ejt.2014.79

41. Van de Vijver B, Wetzel CE, Ector L. Analysis of the type material of *Planothidium delicatulum* (Bacillariophyta) with the description of two new *Planothidium* species from the sub-Antarctic Region. Fottea. 2018; 18(2): 200-211. https://doi.org/10.5507/fot.2018.006

42. Vinocur A, Maidana NI. Spatial and temporal variations in moss-inhabiting summer diatom communities from Potter Peninsula (King George Island, Antarctica). Polar Biol. 2010; 33(4): 443–455. https://doi.org/10.1007/s00300-009-0719-x

43. Zidarova R, Kopalová K and Van De Vijver B. Ten new *Bacillariophyta* species from James Ross Island and the South Shetland Islands (Maritime Antarctic Region). Phytotaxa. 2016; 272(1): 37-62.

44. Filgueiras BK, Tabarelli M, Leal IR, Vaz-De-Mello FZ, Peres CA and Iannuzzi L. Spatial replacement of dung beetles in edge-affected habitats: biotic homogenization or divergence in fragmented tropical forest landscapes?. Divers Distrib. 2016; 22(4): 400-409.

45. Colwell RK. EstimateS: Statistical estimation of species richness and shared species from samples. Available at: http://viceroy.eeb.uconn.edu/EstimateS (last accessed 11 June 2017). 2013.

46. Bitencourt BS and da Silva PG. Forest regeneration affects dung beetle assemblages (*Coleoptera*: *Scarabaeinae*) in the southern Brazilian Atlantic Forest. J Insect Conserv. 2016; 20(5): 855-866.

47. Jost L. Entropy and diversity. Oikos. 2006; 113(2): 363-375.

48. Jost L. Partitioning diversity into independent alpha and beta components. Ecology. 2007; 88(10): 2427-2439.

49. Marcon E and Hérault B. Entropart: An R Package to Measure and Partition Diversity. Available at: http://CRAN.R-project.org/package=entropart (last accessed 11 June 2017). 2014.

50. Manly BF. A method for the estimation of parameters for natural stage-structured populations. Res Popul Ecol. 1997; 39(2): 101-111.

51. Oksanen J, Blanchet FG, Kindt R, Legendre P, O'Hara RB, Simpson GL, Solymos P, Stevens MHH, Wagner H. Vegan: community ecology package. Available at: http://cran.r-project.org/web/packages/vegan/index.html (last accessed 11 June 2017). 2016.

52. Baselga A. Partitioning the turnover and nestedness components of beta diversity. Glob Ecol Biogeogr. 2010; 19(1): 134-143.

53. Baselga A. Separating the two components of abundance-based dissimilarity: balanced changes in abundance vs. abundance gradients. Methods Ecol Evol. 2013; 4(6): 552-557.

54. Baselga A and Orme CDL. Betapart: an R package for the study of beta diversity. Methods Ecol Evol. 2012; 3(5): 808-812.

55. Putzke J and Pereira AB. The Antarctic mosses, Canoas, Brazil: Editorial ULBRA, 196 p. 2001.

56. Majewska R, Gambi MC, Totti CM and De Stefano M. Epiphytic diatom communities of Terra Nova Bay, Ross Sea, Antarctica: structural analysis and relations to algal host. AntarctAntartic Sci. 2013; 25(4): 501-513.

57. Trivelpiece WZ, Hinke JT, Miller AK, Reiss CS, Trivelpiece SG and Watter GM. Variability in krill biomass links harvesting and climate warming to penguin population changes in Antarctica. P Natl Acad Sci USA. 2011; 108(18), 7625-7628.

58. Schaefer CEGR, Simas FNB, Albuquerque-Filho MR, Michel RFM, Viana J. and Tatur HM. Fosfatização: processo de formação de solos na Baía do Almirantado e implicações ambientais. In: Schaefer CEGR Francelino MR, Simas FNB, Albuquerque-Filho MR (Eds.). Ecossistemas costeiros e monitoramento ambiental da Antártica Marítima: Baía do Almirantado, Ilha Rei George [Coastal ecosystems and environmental monitoring of Maritime Antarctica; Admiralty Bay, King George Island](pp. 47-59). 2004. (pp. 47-59). Viçosa:NEPUT-Departamento de Solos.

59. Shmida AVI and Wilson MV. Biological determinants of species diversity. Journal of Biogeography. 1985;1-20.

60. Sutherland DL. Surface-associated diatoms from marine habitats at Cape Evans, Antarctica, including the first record of living *Eunotogramma marginopunctatum*. Polar Biol. 2008; 31(7): 879-888.

61. Mouquet N and Loreau M. Community patterns in source-sink metacommunities. Am Nat. 2003; 162(5): 544-557.

62. Maidana NI, Izaguirre I, Vinocur A, Mataloni G and Pizarro H. Diatomeas en una transecta patago□nico-anta□rtica. Ecolog□ıa Austral. 2005; 15: 159-176.

63. Izaguirre I, Saad JF, Schiaffino MR, Vinocur A, Tell G, Sa□nchez ML, Allende L and Sinistro R. Drivers of phy-toplankton diver- sity in Patagonian and Antarctic lakes across a latitudinal gradient (2150 km): the importance of spatial and environ- mental factors. Hydrobiologia. 2016; 764: 157-170.

64. Schiaffino MR, Unrein F, Gasol JM, Massana R, Balague□V and Izaguirre I. Bacterialcommunity structure in a latitudinal gradient of lakes: the roles of spatial versus environmental factors. Freshw Biol. 2011; 56: 1973-1991.

FIGURE LEGENDS

Figure 1. Map of the location of sampling points where *Prasiola crispa* was collected in Antarctica. Ardley Island (62°12'40.40"S 058°55'38.70"W), Halfmonn Island (62º35'41,5"S 059º55'10,1"W) and King George Island: Copacabana (62°10'38,4"S 058°26'57,3"W), Punta Plaza (62°5'14.69"S 058°23'35,21"W), Steinhoose (62°04'18,9"S 058°22'28,2"W) and Voureal (62º10'2,6"S 058º17’52.0"W). The Scar Antarctic Digital Database was used as background shapefile to produce this image and the authors follow the Licence Terms and Conditions under de CC-BY disclamer. More information can be accessed at https://add.data.bas.ac.uk/repository/entry/show?entryid=f477219b-9121-44d6-afa6-d8552762dc45.

Figure 2 (1-27). Taxa of diatom[illegible] LM. 1. *Achnanthes* sp, 2. *Achnanthidium aff indistinctum,* 3. *Cyclotella meneghiniana,* 4-10. *Cocconeis pinnata* var. *matsii,* 11. *Eunotia aff pseudopaludosa,* 12. *Fragilaria* cf *parva,* 13. *Hantzschia amphioxys,* 14-17. *Luticola amoena,* 18-21. *Luticola austroatlantica,* 22-25. *Luticola muticopsis,* 26. *Luticola olegsakharovii e* 27. *Mayamaea* cf *atomus.* LM scale bar = 10 µm.

Figure 3 (28-60). 28-31. Taxa of diatoms [illegible]M. *Navicula aff perminuta,* 32-34. *Pinnularia australoschoenfelderi,* 35-37. *Pinnularia austroshetlandica,* 38-41. *Pinnularia borealis,* 42-44. *Pinnularia aff microstauron,* 45-48. *Psammothidium germainii,* 49. *Psammothidium papilio,* 50. *Psammonthidium rostrogermainii,* 51-55. *Pseudogomphonema kamtschaticum,* 56-59. *Pteroncola carlinii* e 60. *Thalassiosira gracilis var.expecta.* LM scale bar = 10 µm.

Figure 4 (61-80). 61-63. Taxa of diatoms i[illegible]EM. *Cocconeis pinnata* var. *matsii* , 64-65. *Luticola amoena,* 66-68. *Luticola multicopsis,* 69-71. *Navicula aff perminuta,* 72-73. *Pinnularia austroshetlandica,* 74-75. *Psammothidium germainii,* 76-77. *Pseudogomphonema kamtschaticum* e 78-80. *Pteroncola carlinii.*

Figure 5. Rarefaction curves of the diatom species associated with *Prasiola crispa* for the sampled sites. The vertical line represents the point of comparison between the islands. Confidence intervals were omitted for better visualization. Ard = Ardley; Cop = Copacabana; Hal = Half Moon; PuP = Punta Plaza; Ste = Steinhoose; Vou= Voureal.

Figure 6. Plots of abundance (proportional [pi]) of the diatom species associated with *Prasiola crispa* for the sampled sites. The abbreviations refer to the names of the species in Table 2.

Figure 7. Average dissimilarity between sampling points of the diatom species associated with *Prasiola crispa* for the sampled sites based on the presence / absence of species (A) and abundance (B).

Figure 8. Similarity of Jaccard (A) decomposed into components of substitution (B) and nesting (C), and similarity of Bray-Curtis (D) decomposed into components of balanced variation of abundance (E) and abundance gradients (F) of the diatom community associated with *Prasiola crispa* related to the geographical distance between the sampling sites.

Figure 1

Click here to access/download;Figure;Figure_1.jpg

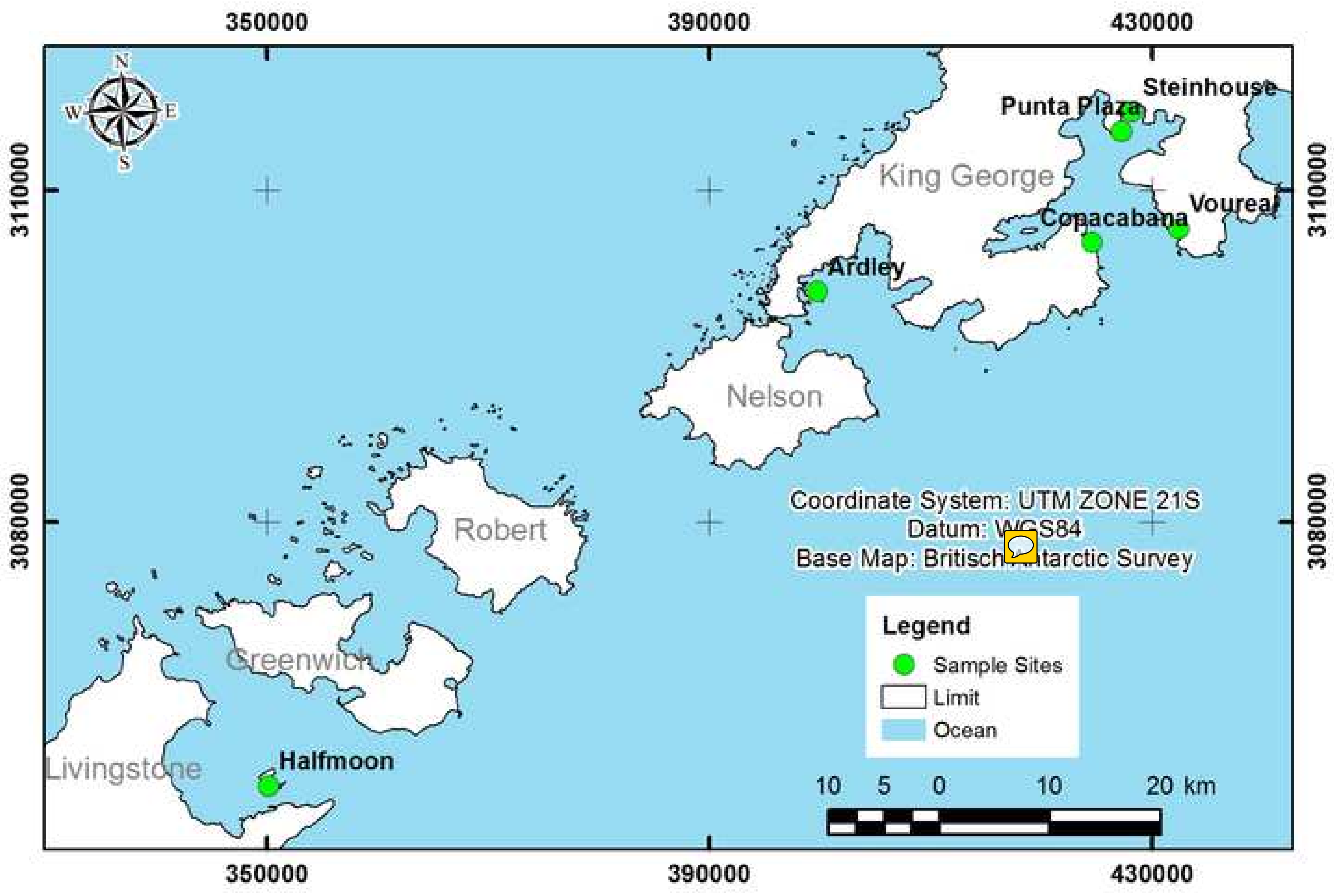

Figure 2

Click here to access/download;Figure;Figure_2.tif

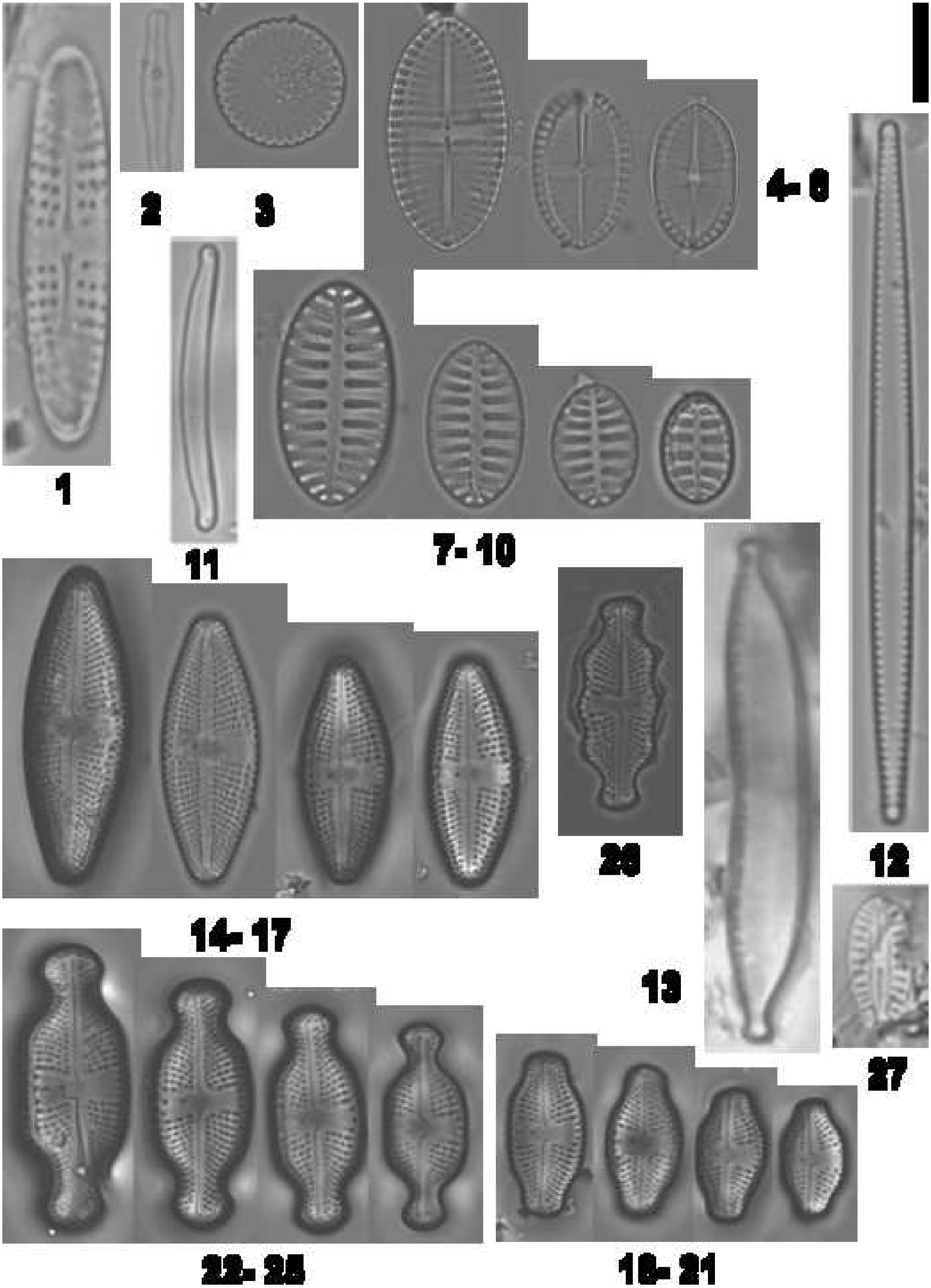

Figure 3

Click here to access/download;Figure;Figure_3.tif

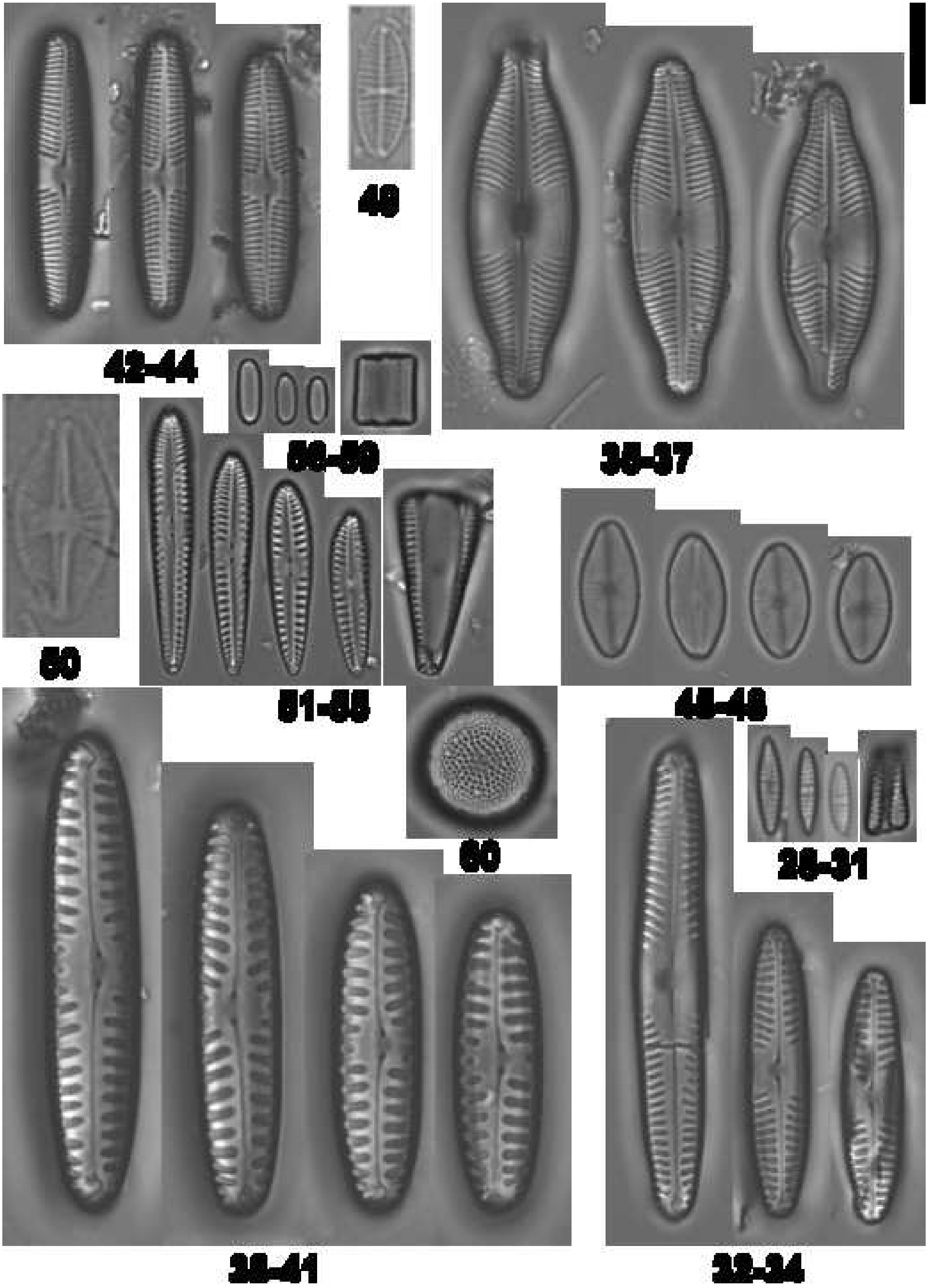

Figure 4

Click here to access/download;Figure;Figure_4.tif

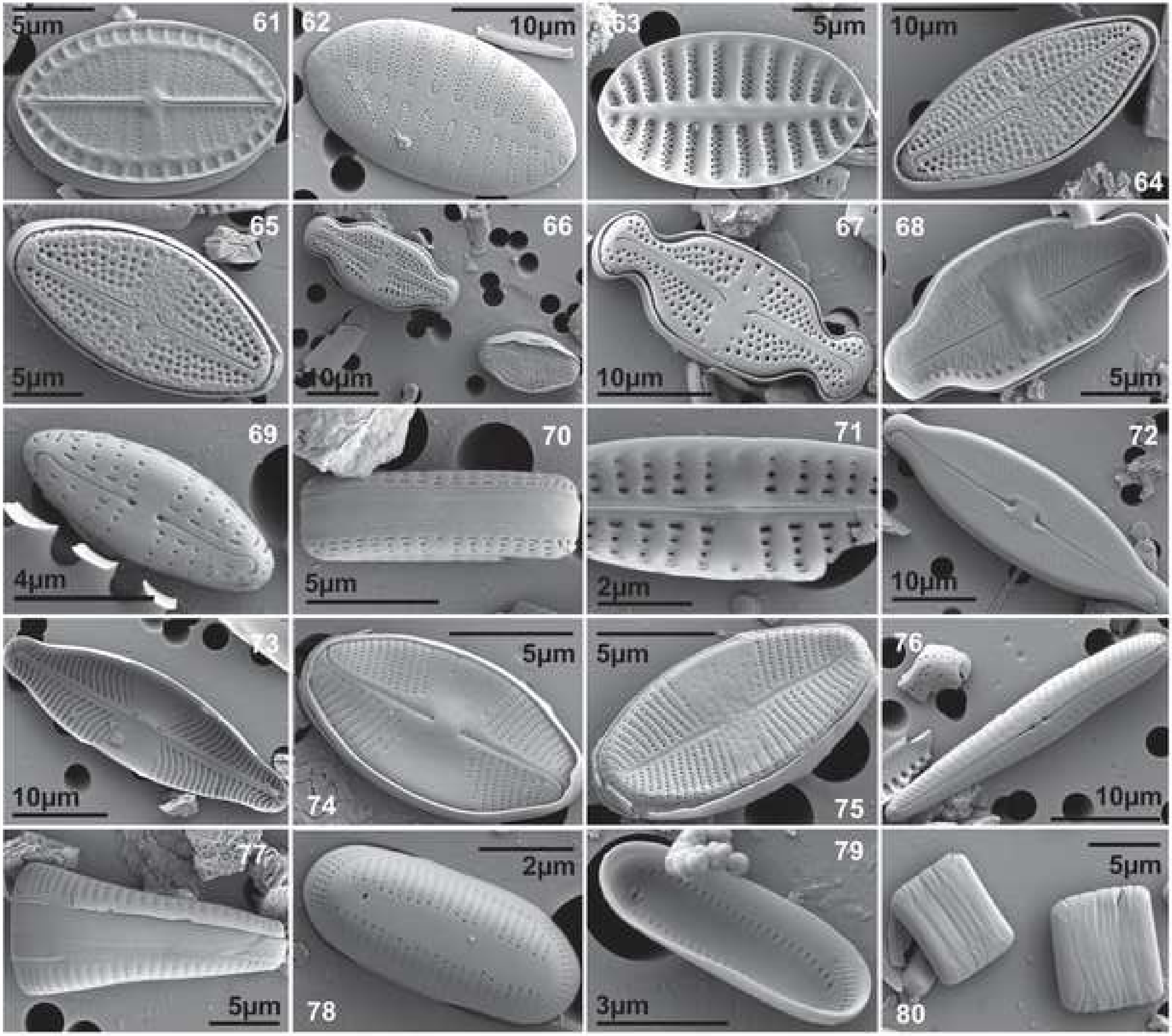

Figure 5

Click here to access/download;Figure;Figure_5.tif

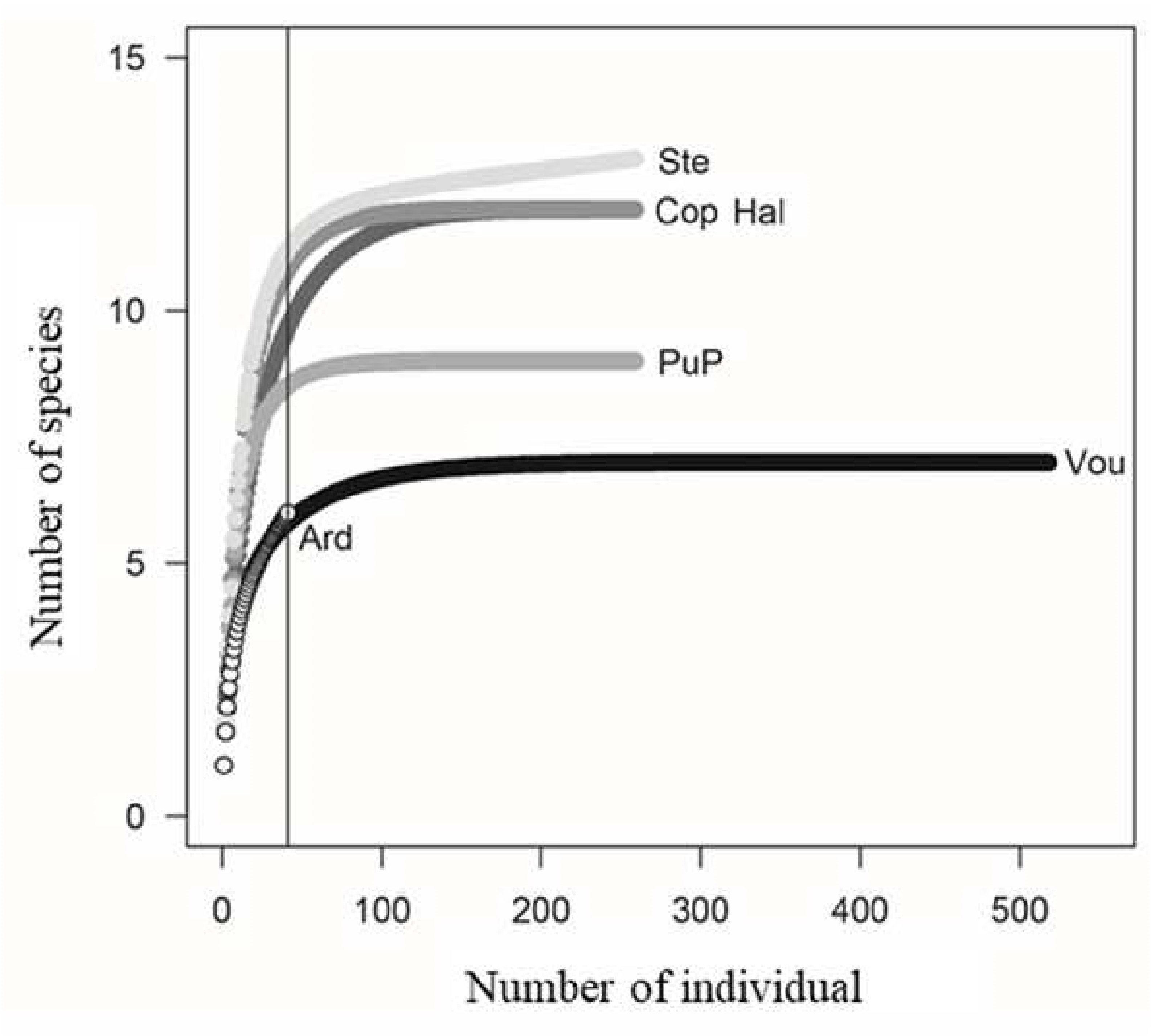

Figure 6

Click here to access/download;Figure;Figure_6.tif

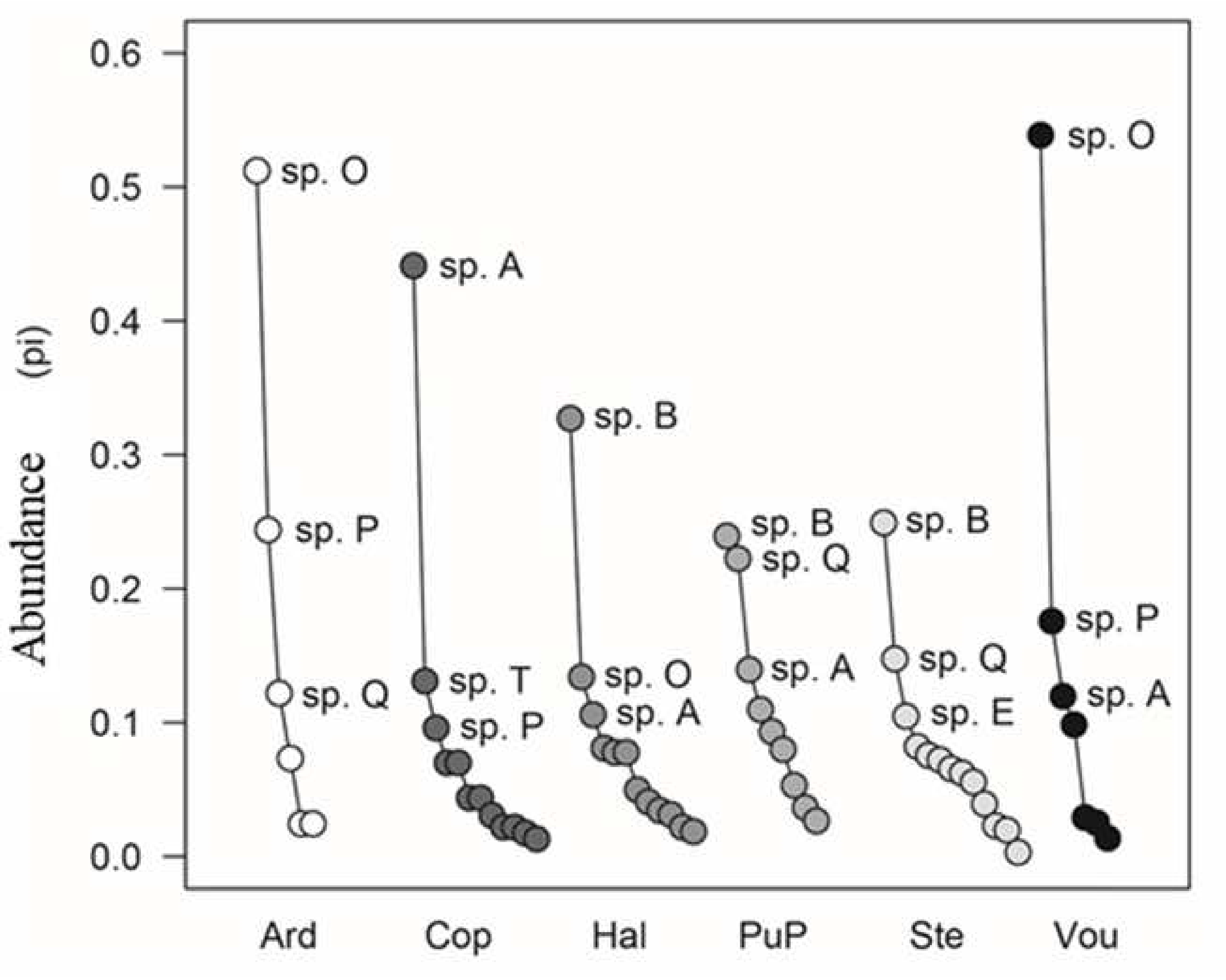

Figure 7

Click here to access/download;Figure;Figure_7.tif

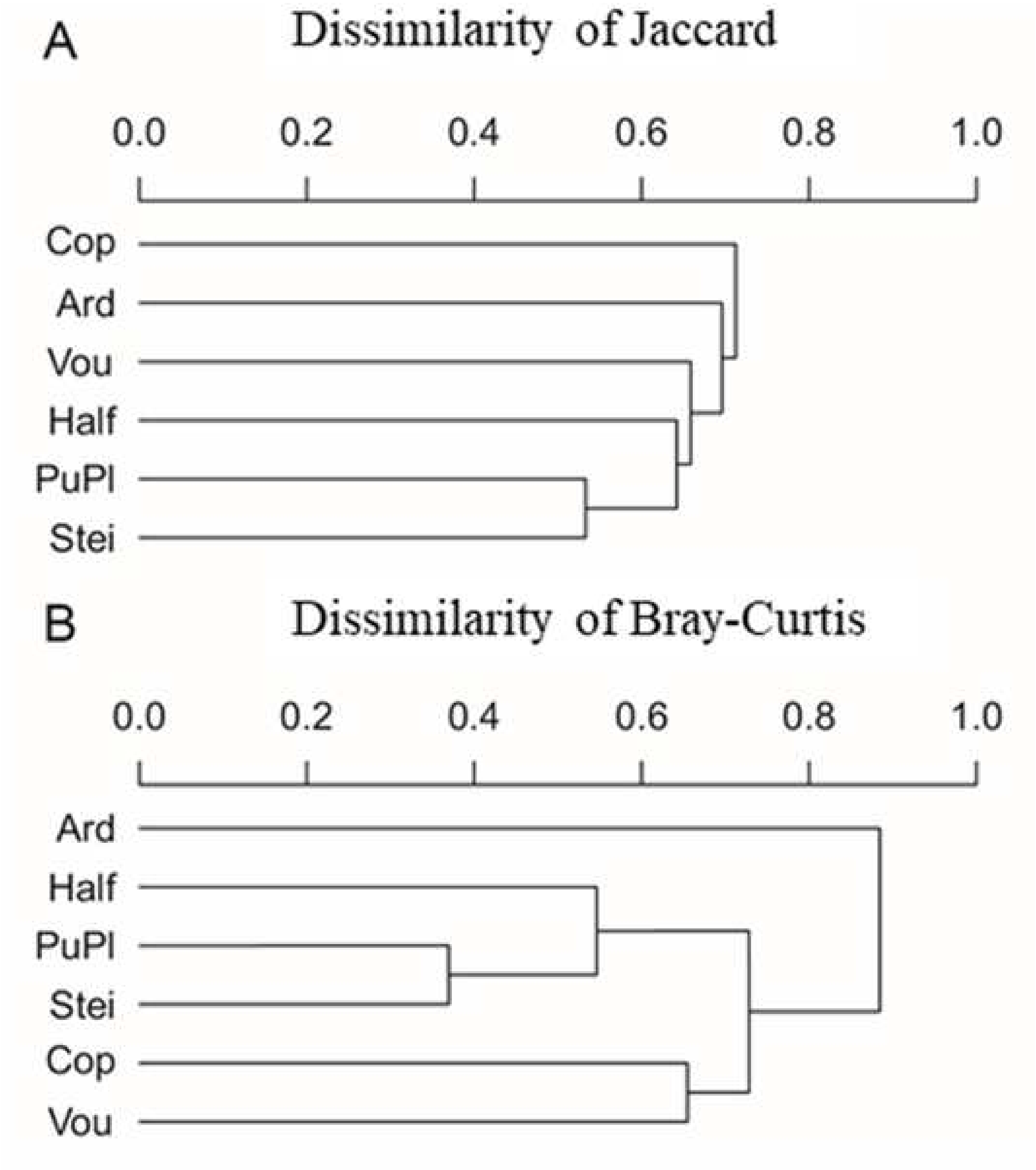

Figure 8

Click here to access/download;Figure;Figure_8.tif

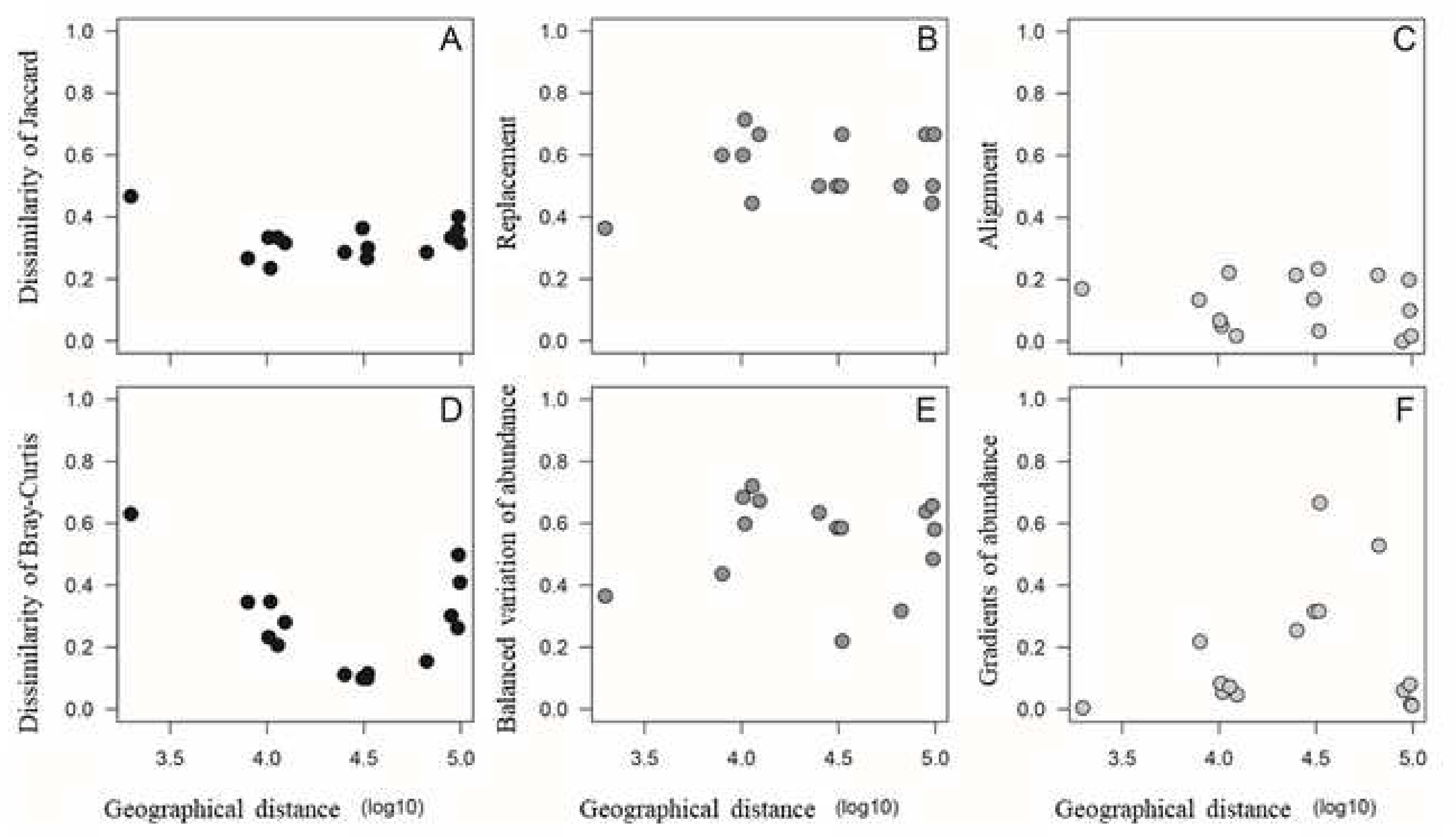

Distribution of aerophilous diatom communities associated with terrestrial green macroalgae in the South Shetland Islands, Maritime Antarctica

Juliana Ferreira da Silva[1]
AntarcticAntarcttic Vegetation Studies Center. Federal University of Pampa. Antonio Trilha Avenue, 1847, 97300-000, São Gabriel, RS, Brazil
Maria Angélica Oliveira
Laboratory of Phycology. Federal University of Santa Maria-. Roraima Avenue, 1000, 97105-900, Santa Maria, RS, Brazil
Raylane Ribeiro da Anunciação
Laboratory of Phycology. Federal University of Pernambuco. Dom Manuel de Medeiros Street, 52171-900, Recife, PE, Brazil
Eduardo Pereira da Silva
AntarcticAntarcttic Vegetation Studies Center. Federal University of Pampa. Antonio Trilha Avenue, 1847, 97300-000, São Gabriel, RS, Brazil
Rodrigo Paidano Alves
Max Planck Institute for Chemistry. Andre Araujo Avenue, 2936, 69067-375, Manaus, AM, Brazil
Adriano Luis Schünemann
AntarcticAntarcttic Vegetation Studies Center. Federal University of Pampa. Antonio Trilha Avenue, 1847, 97300-000, São Gabriel, RS, Brazil
Filipe de Carvalho Victoria*
AntarcticAntarcttic Vegetation Studies Center. Federal University of Pampa. Antonio Trilha Avenue, 1847, 97300-000, São Gabriel, RS, Brazil, filipevictoria@unipampa.edu.br
Margéli Pereira de Albuquerque
National Institute of Antarctic Science and Tecnology – INCT/APA, AntarcticAntarcttic Vegetation Studies Center. Federal University of Pampa. Antonio Trilha Avenue, 1847, 97300-000, São Gabriel, RS, Brazil
and Antônio Batista Pereira
AntarcticAntarcttic Vegetation Studies Center. Federal University of Pampa. Antonio Trilha Avenue, 1847, 97300-000, São Gabriel, RS, Brazil

Running title: Diatoms associated with Antarctic green macroalgae.

* Corresponding Author

ABSTRACT

The establishment of diatom communities depends on environmental factors such as the type of substratesubstrates and geographic conditions that influence the dispersal processes of these organisms. The main goal of this study was to evaluate the similarity between diatom communities associated with the macroalgae *Prasiola crispa* (Lightfoot) Kützing in relation to spatial distance from six sampled sites located in the South Shetland Islands, Maritime Antarctica. The diatom flora associated with *Prasiola crispa* was represented by 23 species distributed in 15 genera. *Pinnularia australoschoenfelderi* Zidarova, Kopalová & Van de Vijver, *LuticolaaustroatlanticaLuticola austroatlantica* Van de Vijver, Kopalová, S.A.Spaulding & EspositoEsposito*multicopsis* (Van Heurck) D.G. Mann, *Luticola amoenaaff beyensiiamoena* Van der Vijver, Kopalová, Zidarova & Levkov Van de Vijver, Ledeganck et Lebouvier, *Pinnularia austroshetlandica* (Carlson) Cleve-Euler and *Psammothidium papilio* (D.E. Kellogg et al.) Kopalová & Zidarova were the most abundant species in ourours samples, which; together they represented 68% of the total number of individuals collected. There is was a great similarity in composition and abundance of the diatom community communities among the sampled points.,Futhermore, many close or distant sampling points have high similarity in the composition and abundance f the diatom community, which resulted in the absence of a linear relationship pattern of relationship with the distance between sampling points. We conclude that distance was not a factor of differentiation of Antarctic diatom communities associated with terrestrial green macroalgae. Suggesting This suggests that Antarctic environments may have unique characteristics with homogeneous abiotic factors, at least related in relation to this substrate.

Keywords: Algae, Antarctic, Biogeography, Aerophilous diatomsDdiatoms, Taxonomy.

INTRODUCTION

The diatoms species diversity of the Antarctic is limited by extreme conditions, and studies have led to the conclusion that not all Antarctic islands share a similar flora, although the overall diversity of this flora on the continent is limited compared to tropical and subtropical regions [1].] (Hamsher et al. 2016). Furthermore, many of the diatoms taxa recently described are endemic to Antarctica and not cosmopolitan as previously reported [2,3].] (Van de Vijver et al. 2005, Kopalová et al. 2014). Regarding the study of diatom communities, in recent years, a more refined taxonomy has revealed a large number of new species for in Antarctica [3-16].Antarctica [4-13] (Kochman-Kędziora et al. 2016, 2017, 2018, Kociolek et al. 2017, Kopalová et al. 2014, Van de Vijver et al. 2010, 2011a, 2011b, 2013, Van de Vijver et al. 2016, 2018, Zelazna-Wieczorek and Olszynski 2016, Zidarova et al. 2012, 2016).

Among biological substrates where microalgal communities may grow, terrestrial macroscopic algae (–though unexpected at first)— may be included. The class Trebouxiophyceae includes a group of morphologically heterogeneous eukaryotic green algae that occur mainly in soil and continental waters. The genus *Prasiola* Meneghini belongs to this class and includes marine, terrestrial and continental species. *Prasiola crispa* (Lightfoot) Kützing is a terrestrial species of Antarctica, which usually grows on moist soils that are fertilized by the guano of birdsbird guano, being more abundant inside and around penguin colonies. Theseis macroalga tolerates repeated cycles of freezing and thawing during the year, as well as high levels of UV radiation during the summer [17].1 4] (Guiry and Guiry 2016). At the micro-scale level, the substrate of periphytic communities may provide more than an inert surface, since its physical and chemical characteristics can influence the composition of the community and algal biomass [18-20].15-17] (Murdock and Dodds 2007, Bergey 2008, Santos and Ferragut 2013).

Studies that address distribution patterns often document a decrease in species similarity as spatial distance increases between communities [21-23].18-20] (Nekola and White 1999, Wetzel et al. 2012, Goldenberg Vilar et al. 2014). This reduction of similarity is attributed to some major factors such as environmental conditions and species

Commented [A1]: Perhaps it may be more suitable to use ‘overall diversity’

Commented [A2]: While both ‘diatoms taxa’ and ‘diatom taxa’ are used in the literature, the latter is more common and seems more grammatically sound.

Commented [A3]: This may not be necessary, since you already mention ‘recently described’ earlier in the sentence.

Commented [A4]: The use of ‘-‘ can seem a little uncommon. Perhaps you could use
“terrestrial macroscopic algae (though unexpected at first) may”
Or
“terrestrial macroscopic algae, though unexpected at first, may”

Commented [A5]: I suggest you can change to ‘These macroalgae’ or ‘This macroalgal species’

dispersal processes. Spatial distance is a factor that influences,~~For~~ the larger the distance between sites, the larger are the environmental variations and, therefore, the the difference between species tends to be also larger. Spatial distance is a factor that influences the displacement of species across smaller distances, once that this factor increases the chances of survival, different from migration over greater distances [21].~~[18] (Nekola and White 1999).~~ The main goal of this study was to evaluate the similarity between diatom communities associated ~~to~~ with~~*Prasiola*~~ P. *crispa* spatially distributed in six sites located in the South Shetland Islands, Antarctica. The ~~hypothesis~~ proposed hypothesis is that the larger the spatial distance between sampling points, the smaller the similarity of ~~the~~ diatom communities~~ycommunity~~ associated with *P. crispa*.

MATERIAL AND METHODS

During the Brazilian Antarctic Expeditions XXXIII (2014-2015) and XXXIV (2015-2016) (Fig. 1), samples of ~~*Prasiola*~~ P. *crispa* where obtained from Ardley Island, Halfmoon Island and King George Island (Copacabana, Punta Plaza, Steinhoose and Voureal), in the Antarctica Maritime. All collections were authorized by the Secretariat of the Interministerial Commission for Sea Resources (SECIRM) and endorsed by the Brazilian Government's Ministry of the Environment, under the activities regulated by the Brazilian Antarctic Program (PROANTAR).

Table 1– Sample site coordinates.

Sample Site	Latitude	Longitude
Ardley Island	62°12'40.40"S	058°55'38.70"W
Halfmonn Island	62°35'41.5"S	059°55'10.1"W
King George Island: Copacabana	62°10'38.4"S	058°26'57.3"W
King George Island: Punta Plaza	62°5'14.69"S	058°23'35.21"W
King George Island: Steinhoose	62°04'18.9"S	058°22'28.2"W
King George Island: Voureal	62°10'2.6"S	058°17'52.0"W

The samples of *P. crispa* were stored in sealed plastic bags and frozen for further analysis in the Laboratory of Phycology of the Federal University of Santa Maria (UFSM), Rio Grande do Sul, Brazil. To prepare the material, cell contents and organic matter of the samples were removed by oxidation with hydrogen peroxide (H_2O_2) and potassium permanganate ($KMnO_4$) [24].~~21] (Kelly et al. 1998).~~ After washing with distilled water, permanent slides were mounted using Naphrax (refractive index of 1.74, Brunel Microscopes Ltd, Chippenham, Wiltshire, United Kingdom).~~.),~~ Qualitative and quantitative analyses were carried out under a Leica DM750 optical microscope. ~~The quantitative~~ Quantitative ~~analysis~~ analyses ~~was~~ were performed with approximately 80 valves for each sample, because the abundance is very small in ~~this~~ the substrate. Thus, i~~I~~t was necessary to ~~make~~ prepare a lot of permanent

Commented [A6]: This sentence may not be quite clear to the reader. Perhaps you mean to say:
"Spatial distance is an influencing factor, whereby larger distance between sites are related to larger environmental variations, and, subsequently, differences between species tend to also be larger"

Commented [A7]: This sentence may also be a little unclear to the reader. Do you mean to say:
"Spatial distance is also a factor that influences the displacement of species across smaller distances; movement in these distances increases the chances of survival, and is different to migration over greater distances"

slides to make observations under this criteriaobserve near this amount. Taxa were identified following Round et al. [25].[22] (1990). A specific bibliography to for identifyingidentify diatoms from polar environmental was used [1, 8-11; 14-15, 26-43].was used for identification, for example, Al-Handal et al. (2010); Bonaventura et al. 2006; Esposito et al. 2008; Hamsher et al. 2016; Kawecka et al. 1998; Kochman-Kędziora et al. 2018; Kopalová 2012; Kopalová et al. 2011, 2013, 2015; Lobo et al. 1998; Luo et al. 2016; Mataloni et al. 2005; Pizarro and Vinocur 2000; Procopiak and Fernandes 2003; Van de Vijver et al. 2008, 2009, 2011, 2013, 2018; Van de Vijver and Beyens 1996; Van de Vijver and Kopalová 2014; Van de Vijver and Mataloni 2008; Van de Vijver and Zidarova 2011; Vinocur and Izaguirre 1994; Vinocur and Maidana 2010; Zidarova 2010; Zidarova et al. 2012 and Zidarova et al. 2016.

For data analysis, the spatial distance in meters metres between each pair of sampling sites was calculated based on the registered geographical points. Thus, a geographic distance matrix (Table 21) was constructed for further comparison with using the matrices from biological similarity of relative abundance (abundance of individuals by taxa) (Table 32) and species composition (presence and absence of taxa in points).

The estimation of species richness was based on the a simple arithmetic of the estimators, without considering theirconsideration of standard deviations [44].23] (Filgueiras et al. 2016). The sampleSsample sufficiency was calculated based on the percentage of values observed in relation to the estimated values. In order to compare the species richness among the sample points, individual rarefaction curves were constructed, and compared in the lowest abundance value among them (N = 41), in order to correct for differences in size and sample effort. For this purpose, the abundance data of each species were used for each sampling point. The EstimateS program (Department of Ecology & Evolutionary Biology, University of Connecticut, Storrs, USA) was used for these analyzesanalyses, with 100 randomisations of the data [45].24] (Colwell 2013). Rank-abundance diagrams were constructed to describe and compare the distribution of abundance and dominance of species at each sampling site using relative abundance data [46].25] (Bitencourt and Da Silva 2016). To describe these distributions numerically, the Pielou equitability index was adopted, based on the Shannon diversity index.

Table 2. Distance table in kilometers between sampling points where *Prasiola crispa* was collected in Antarctica.

Sample sites	Ardley	Copacabana	Halfmoon	Punta Plaza	Steinhouse	Voureal
Ardley	0					
Copacabana	25.17	0				
Halfmoon	66.76	89.14	0			
Punta Plaza	31.07	10.43	97.19	0		
Steinhouse	32.74	12.37	99.00	1.98	0	
Voureal	33.13	7.96	96.46	10.20	10.55	0

Commented [A8]: Perhaps you mean to say 'to make observations under this criteria'.?

Commented [A9]: Please change to
"A specific bibliography for identifying diatoms from polar environments was used"
Or
"Specific references for identifying diatoms from polar environments were used"

Commented [A10]: British English

Table 3. List of all observed species with their acronyms and abundance in the samples from: Ard= Ardley; Cop= Copacabana; Hal=Half Moon; PuP= Punta Plaza; Ste= Steinhoose; Vou= Voureal. Species	Acronyms	Sample points					
		Ard	Co	Hal	Pu	Ste	Vo
Luticola amoena Van der Vijver, Kopalová, Zidarova & Levkov	A	0	0	0	0	5.	0
Luticola austroatlantica Van de Vijver, Kopalová, S.A. Spaulding & Esposito	B	0	0	0	0	2.	0
Luticola muticopsis (Van Heurck) D.G. Mann	C	0	0	0	0	1.	1.
Achnanthes sp.	D	0	0	0	2.	0	0
Achnanthidium aff *indistinctum* Van de Vijver & Kopalová	E	0	1.	0	0	1	0
Cyclotella meneghiniana Kützing 184	F	0	1.	0	0	0	0
Cocconeis pinnata var. *matsii* Al-Handal, Riaux-Gobin & Wulff	G	2.	4	1	1	7.	1
Eunotia aff *pseudopaludosa* Van de Vijver, de Haan & Lange-Bertalot	H	0	0	3	2	2	2.
Fragilaria cf *parva* Tuji & D.M. Williams	I	2.	4.	1.	3.	0.	0
Hantzschia amphioxys f. *muelleri* Ts. Ko-Bayashi	J	7.	4.	0	0	0	0
Pteroncola carlinii Almandoz & Ferrario 2014	K	0	0	0	8.	7.	0
Mayamaea cf *atomus* (Kützing) Lange-Bertalot 1997	L	0	0	4.	0	0	0
Navicula aff *perminuta* Ǿstrup 1913	M	0	2.	0	0	0	0
Pinnularia aff *microstauron* (Ehrenberg) Cleve 1891	N	0	3.	3.	0	0	0
Pinnularia australoschoenfelderi Zidarova, Kopalová & Van de Vijver	O	5	0	1	0	0	5
Pinnularia austroshetlandica (Carlson) Cleve-Euler	P	2	9.	0	4.	6.	1
Pinnularia borealis Ehrenberg sensu lato	Q	1	0	3.	9.	1	0
Psammonthidium rostrogermainii Van de Vijver, Kopalová & Zidarova	R	0	2.	0	0	6.	0
Psammothidium germainii (Manguin) Sabbe	S	0	0	7.	0	3.	0
Psammothidium papilio (D.E. Kellogg et al.) Kopalová & Zidarova	T	0	1	2.	7.	8.	9.
Pseudogomphonema kamtschaticum (Grunow) L.K.Medlin, 1986	U	0	6.	4.	0	0	2.
Luticola olegsakharovii Zidarova, Levkov & Van de Vijver	V	0	0	8.	2	0	0
Thalassiosira gracilis var.expecta G. Fryxell & Hasle 1979	X	0	7.	7.	0	0	0

The diversity was partitioned into the alpha, beta and gamma components using Hill's numbers [47,48]~~26,27]~~ ~~(Jost 2006, 2007)~~ with the "entropart" package [49]~~28]~~ ~~(Marcon and Herault 2014)~~ ~~of~~ in the R program (R Foundation for Statistical Computing, Vienna, Austria) (R Core Team 2017), with the approach of multiplicative partitioning of diversity.

Jaccard and Bray-Curtis dissimilarity coefficients were used to calculate dissimilarity between pairs of sampling points based on incidence and abundance data. An array of geographical (Euclidean) distances between

points was derived from the longitude and latitude coordinates. Subsequently, the Jaccard and Bray-Curtis distance matrices were regressed with the geographic distance matrix using linear regressions -[50].29] (Manly 1997).

The standard coefficients of these regression models will bewere used as measures of the decay rate when comparingin the similarity as a function of the geographical distance between points [22]. [30] (Wetzel et al. 2012). In addition, biotic distance data were correlated with the geographic distance matrix in order to corroborate the relationship probability values based on 10.000 permutations using the Mantel test [23].31] (Goldenberg Vilar et al. 2014). Statistical analyzes analyses were performed in the R program using the "vegan" packages [51]32] (Oksanen et al. 2016) and "betapart" [52] packages, and33] (Baselga 2010) visually shownvisualised through dendrograms also Aalso using the "vegan" package [51] in34] (Oksanen et al. 2016) oin R environment.

In order to verify which component of the beta diversity mostly predominantly contributed to the dissimilarity between sampling points, species presence/absence dissimilarity was decomposed into substitution (turnover) and nestedness [52]33] (Baselga 2010) components, while abundance abundance-based dissimilarity was decomposed into the components of abundance balanced variation and abundance gradients [53].35] (Baselga 2013). For this, Jaccard and Bray-Curtis dissimilarity coefficients were used on in the "betapart" package [54]36] (Baselga and Orme 2012) of theimplemented in the R program. The matrices of beta diversity components based on presence–/–absence and abundance were also associated to the geographic distance matrix, as previously described, in order to verify which dissimilarity component was related to the spatial configuration of the sampling sites.

The protocols used for the present research study could can be accessedacessed in through protocols.protocol-io (dx.doi.org/10.17504/protocols.io.6tcheiw).

RESULTS AND DISCUSSION

The diatom flora associated with *Prasiola crispa* at the six sampling sites was were represented by 23 species (Fig. 2-4) distributed in 15 genera. Two species are typical of marine environments: *PseudogomphonemaPseudoghomphonema* *kamtschaticum*— and(Grunow) LKMedlin, *Cocconeis pinnata* var. *matsii*and*matsii* and *Pteroncola carlinii*. Almandoz & Ferrario. The presence of these species in these terrestrial sites is probably related with to their proximity to the sea.

Comparing the rarefied richness of the islands, in order to correct the for differences in sample size differences between sitesbetween them, it was possible to observeWe observed that Steinhoose and Halfmoon had higher species

Commented [A11]: This might not be clear to the reader. Is it a term commonly used in this model. Or perhaps you mean to say “used as measures of the decay rate when compariing similarity as a function of geographical distance between points”?

Commented [A12]: Do you mean to say ‘were also tested for association with the’?

Commented [A13]: May need a reference/citation if you state ‘as previously described’.

Formatted: Left, Indent: First line: 0"

Commented [A14]: Do you mean to say ‘we observed’

richness, even when comparing the same number of individuals (~~smaller~~ total abundance ~~among them;~~ = 41 in both sites) (Fig. 5). Intermediate richness was observed in Copacabana and Punta Plaza, ~~while,~~and even though Voureal~~it~~ had the largest number of individuals, ~~Voureal~~ this site had ~~a rarefied richness~~ similar rarefied richness to that of Ardley, which had the lowest number of species and individuals (Fig. 5).

The most abundant species were *Pinnularia australoschoenfelderi* ~~Zidarova, Kopalová & Van de Vijver~~ (20.0%), *Luticola austroatlantica~~multicopsis~~austroatlantica* ~~(Van Heurck) D.G. Mann Morphotype I~~ (15.5%), *Luticola amoena~~aff beyensii~~amoena* ~~Van de Vijver, Ledeganck et Lebouvier~~ (15.3%), *Pinnularia austroshetlandica* ~~(Carlson) Cleve-Euler (~~9.2%) and *Psammothidium papilio* ~~(DE Kellogg et al.) Kopalová & Zidarova (~~8.0%), which altogether~~together~~ represented 68% of the total number of individuals collected. There was a difference ~~between~~ in the most abundant species ~~in~~ between each sampling site (Fig. 6). The dominance pattern in Steinhoose and Punta Plaza, the ~~pair of~~two areas closest to each other in ~~the~~ this study, was similar, with high abundance of ~~*Luticola*~~ *L. austroatlantica~~multicopsis~~austroatlantica* ~~(Van Heurck) D.G. Mann Morphotype I~~ and *Pinnularia borealis*. ~~Ehrenberg sensu lato.~~ These two areas also had the highest Pielou equitability values (Punta Plaza: Pielou = 0.91, Steinhoose: Pielou = 0.89). However, ~~*Luticola*~~ *L.~~multicopsis~~ austroatlantica*~~(Van Heurck) D.G. Mann Morphotype I,~~ was also the most abundant at the other extreme of the distance gradient in Halfmoon (Pielou = 0.86). ~~*Pinnularia*~~ *P. australoschoenfelderi* ~~Zidarova, Kopalová & Van de Vijver~~ dominated in Ardley (Pielou = 0~~,~~.73) and Voureal (Pielou = 0~~,~~.71), while ~~*Luticola*~~ *L. amoena~~aff beyensii~~amoena* ~~Van de Vijver, Ledeganck et Lebouvier~~ dominated in Copacabana (Pielou = 0.76)~~-~~. This species was also among the three most abundant species in Halfmoon, Punta Plaza and Voureal.

The diversity partition demonstrated that true diversity based on species richness (greater weight on rare species), Shannon's entropy (similar weight on common and rare species), and Simpson index (greater weight on dominant species) showed similar response patterns, with higher values in Steinhoose (Table 4~~3~~). The alpha diversity was approximately ~~10~~ ten species for species richness ~~and~~; this analysis further showed that the true beta diversity of Shannon (1D) was lower than the richness based on species richness (0D) and based on the inverse concentration of Simpson (2D). This shows that differences between sampling sites occurred due to the contrast between the most abundant and the rarest species ~~of~~ at each site (Table 3). The beta diversity values for all orders were close to 2, indicating that there are at least two large groups of species with high substitutions~~substitution~~.

Commented [A15]: This might not be quite clear to the reader. Do you mean to say ‘(total abundance = 41 in both sites)’?

Commented [A16]: When you repeat a species name you can abbreviate the Genus to a single letter.

Commented [A17]: It would be better to specify the species here to avoid confusion e.g. *‘L.amoena’*

Commented [A18]: Do you mean to say ‘Simpson index’?

Commented [A19]: Do you mean ‘of the Shannon index’?

Commented [A20]: Do you mean ‘of the Simpson index’?

Commented [A21]: Perhaps you can use ‘two’?

Table 4. Multiplicative partitioning of the diatom species diversity associated with *Prasiola crispa* for the sampled sites. Ard = Ardley; Half = Halfmoon; Cop = Copacabana; Pupl = Punta Plaza; Stei = Steinhoose; Vou = Voureal; true beta diversity of Shannon (^{1}D); species richness (^{0}D); concentration of Simpson (^{2}D).

	Islands						Components of diversity		
	Ard	Half	Cop	PuPl	Stei	Vou	A	β	γ
^0D	6	12	12	9	13	7	10	2.3	23
^1D	3.73	8.45	6.68	7.31	9.75	3.95	6.38	1.92	12.26
^2D	2.91	6.17	4.22	6.32	7.92	2.88	4.44	2.01	8.92

Total dissimilarity based on the presence/absence and abundance of species between the islands was 80.7% and 80.5%, respectively. Similarly, ~~analyzes~~ analyses of parity-by-pair dissimilarity (both presence/absence and abundance based), showed similar patterns of response, but no formation of distinct groups (Fig. 7). The high similarity between the nearest sites (Steinhoose and Punta Plaza), with the most distant site (Halfmoon), for presence/absence as well as for number of individuals, should be emphasized. Copacabana had the highest average dissimilarity with the other sampling sites based on species composition (Fig. 7A), while Ardley had the greatest dissimilarity based on abundance of individuals (Fig. 7B).

The decomposition of beta diversity based on the presence/absence of species showed that the species substitution component was the most important relative to dissimilarity among the sampling sites (73.1%). The relative importance of the nesting component was only 7.6%, corroborating the beta diversity values slightly higher than ~~2 of the beta diversity,~~ two shown by the partitioning analysis of diversity in the alpha, beta and gamma components. These results were strongly influenced by the observed species richness in each sampling site, as well as the high number of exclusive species (26.1% of the total species). In addition, only 26.1% of the species occurred in more than 50% of the sites.

Community similarity between sampling sites was not related to the geographical distance between them, based on ~~the~~ incidence (t = -0.509, p = 0.619) and abundance (t = -1.041, p = 0.3167) (Fig. 8A and D). Species substitution components (t = 0.610, p = 0.553) and nesting (t = -0.403, p = 0.693) of incidence-based beta diversity were also not related to geographical distance (Fig. 8B and C). Similarly, beta diversity components based on the abundance from balanced variation of abundance (t = 0.180, p = 0.860) and abundance gradients (t = 0.654, p = 0.525)

> **Commented [A22]:** Perhaps you can use 'two'?

> **Commented [A23]:** It seems coincidental that both of these values are 26.1%. Please double check these values to make sure whether they are the same or not.

were not related to the geographical distance between sampling sites (Fig. 8~~-~~E~~, ~~ and F). The Mantel tests, based on 10~~,~~000 permutations, corroborated the probability values for this lack of relationship between the similarity matrix and ~~the~~ dissimilarity components with the between-sites geographical distance. The lack of a positive or negative similarity relationship ~~to the~~with geographical distance occurred due to the high similarity in composition and abundance among the most distant sampling points. In other words, even very close or very distant ~~places~~ sites have ~~a~~ high similarity in diatom composition and abundance, which resulted in the absence of a linear relationship pattern ~~of~~ with distance ~~relationship~~.

Prasiola crispa is found in drainage lines in Antarctica, and because it is ornithocoprophilous, presents considerable biomass mainly around penguin colonies [55~~], ~~]. Thus, this~~37] (Putzke and Pereira 2001), which~~ makes nutrient availability for both the green algae and~~alga an~~ the surrounding diatom community higher, ~~if compared~~~~relative to other habitats like~~when compared to e.g. ~~to~~ epilithic habitats~~, for example~~. The lack of differentiation between the communities with geographic distance could also be attributed to the lower climatic variation in Antarctica compared to tropical and subtropical regions.~~Another important factor for not differentiating the communities with the geographic distance is that the climatic variation in Antarctica is low compared to tropical and subtropical regions~~. In ~~the~~ a ~~work done~~previous study ~~by Majewska et al. [56], with~~of~~38], with~~ three marine red macroalgae in Newfoundland Bay, Antarctica [56]~~,~~ the biophysical-chemical characteristics of each sampling site affected the diatom communities more ~~differently~~ markedly than the different species of host macroalgae or even depth of sampling. Moreover, t~~T~~he diversity of the epiphytic community of diatoms associated with red macroalgae collected near the sea ice of Cape Evans, Antarctica, showed that species diversity decreased as the depth below the sea ice increased, whereby~~where~~ the dominant taxa ~~also~~ had also~~has~~ changed in relation to depth [~~5939~~59-61].

~~Results~~ Our study showed that even with ~~the~~ marked distance between sampling points, there was ~~a~~ high similarity in the composition and abundance of the diatom ~~community~~communities, which resulted in the absence of a linear relationship pattern ~~of relation~~ with ~~the~~ distance. However, it was possible to demonstrate that regarding dominant species, the closest sampling sites were more similar. Thus, the inexpressive differentiation of the ~~community~~ communities showed that *P. crispa* as a substrate seems to be an~~d~~ important factor for the selection of the existing epiphytic community.

~~Increasingly in the literature,~~There have been an increasing number of studies reporting changes in the community of Antarctic organisms ~~are being portrayed~~ due to global warming. Some penguin communities are

Commented [A24]: I divided this sentence as it was a little lengthy.

Commented [A25]: Alternatively, you can use 'when compared to e.g. epilithic habitats'.

Commented [A26]: Perhaps you mean to say
"The lack of differentiation between the communities with geographic distance could also be attributed to the lower climatic variation in Antarctica compared to tropical and subtropical regions."

Or
"Another important factor related to the lack of differentiation among communities with geographic distance is that the climatic variation in Antarctica is low compared to tropical and subtropical regions."

experiencing population declines as a result of rising temperatures [57], and~~(Trivelpiece et al, 2011)~~, moss communities are increasing on King George Island, colonizing fields in uncovered areas due to shrinking glaciers [58]~~.(Schaefer et al ., 2004)~~. Decreases in microbial diversity patterns has also been reported in regions of Patagonian and other Antarctic lakes, mainly found to decrease in diatom diversity [62], as well with evidence for the decline of phytoplankton [63], and bacterioplankton [64]. Such aspects lead us to reflect on how much the diatom communities, as well as the substrate of the present study (~~, *Prasiola*~~ *P. crispa*)*cripa*~~,~~ can be affected, ~~after all, the species~~since *P. crispa* grows in humid places with high nutrient input. If we ~~have the eleminition of~~eliminate these factors, we ~~will~~ would also affect the existence of this macroalgae species and, consequently, that of the associated aerophytic diatom community. Although we have not observed anyan exclusive ~~community~~ communities present in this substrate, we believe that by affecting the ~~permanence~~stability of *P. crispa* in terrestrial wetlands, we ~~will~~ would also affect the dispersal of associated diatoms~~acea. inclusive.~~

Commented [A27]: A more suitable word might be 'stability'

Commented [A28]: Do you mean to say 'associated diatoms'?

ACKNOWLEDGMENT

~~To~~ We acknowledge the~~To~~ Coordination of Improvement of Higher-Level Personnel (CAPES)~~, National Council for Scientific and Technological Development (CNPq),) and the National Institute of Antarctic Science and Technology for Environmental Research (INCT-APA)~~ for the scholarship granted to the first author~~doctoral and scientific productivity grants~~. The authors would also like to thank the brazilian Ministry of the Environment and the Inter-Ministerial Secretariat for Sea Resources (SECIRM) for the authorizations granted for field collection activities under the Brazilian Antarctic Program.

AUTHOR CONTRIBUTIONS

Conceptualization: Juliana Ferreira da Silva, Maria Angélica Oliveira, Antonio Batista Pereira

Data curation: Juliana Ferreira da Silva, Filipe de Carvalho Victoria, Margéli Pereira de Albuquerque, Eduardo Pereira da Silva, Raylane Ribeiro da Anunciação

Formal analysis: Juliana Ferreira da Silva, Eduardo Pereira da Silva, Raylane Ribeiro da Anunciação, Rodrigo Paidano Alves, Maria Angélica Oliveira

Funding acquisition: Antonio Batista Pereira

Methodology: Juliana Ferreria da Silva, Adriano Luis Schunemann, Maria Angélica Oliveira

Resources: Juliana Ferreria da Silva, Adriano Luis Schunemann

Visualization: Juliana Ferreira da Silva, Adriano Luis Schunemann

Writing – original draft: Juliana Ferreira da Silva, Margéli Pereira de Albuquerque, Rodrigo Paidano Alves, Filipe de Carvalho Victoria, Maria Angélica Oliveira.

Formatted: Indent: First line: 0"

REFERENCES

Commented [A29]: I've made adjustments to ensure are formatting styles are consistent between all references. In addition, I've made suggestions for abbreviated journal names to be consistent throughout.

1. Hamsher S, Kopalova K, Kociolek JP, Zidarova R and Van De Vijver B. The genus *Nitzschia* on the South Shetland Islands and James Ross Island. Fottea. 2016; 16(1): 79-102.

2. Van De Vijver B, Gremmen NJ and Beyens L. The genus *Stauroneis* (*Bacillariophyceae*) in the Antarctic region. J Biogeogr. 2005; 32(10): 1791-1798.

3. Kopalova K, Ochyra R, Nedbalova L and Van De Vijver B. Moss-inhabiting diatoms from two contrasting Maritime Antarctic islands. Plant Ecol Evol. 2014; 147(1): 67–84.

4. Kochman-Kedziora N, Noga T, Zidarova R, Kopalová K and Van DE Vijver B. *Humidophila komarekiana* sp. nov.(*Bacillariophyta*), a new limnoterrestrial diatom species from King George Island (Maritime Antarctica). Phytotaxa. 2016; -272(3): 184-190.

5. Kochman-Kedziora N, Noga T, Van De Vijver B and Stanek-Tarkowska J. A new *Muelleria* species (*Bacillariophyta*) from the Maritime Antarctic Region. Fottea. 2017; 17(2): 264-268.

6. Kochman-Kedziora N, Pinseel E, Rybak M, Noga T, Olech M And Van De Vijver B. *Pinnularia subcatenaborealis* sp. nov.(*Bacillariophyta*) a new chain-forming diatom species from King George Island (Maritime Antarctica). Phytotaxa. 2018; 364(3): 259-266.

7. Kociolek JP, Kopalová K, Hamsher SE, Kohler TJ, Van De Vijver B, Convey P and Mcknight DM. Freshwater diatom biogeography and the genus *Luticola*: an extreme case of endemism in Antarctica. Polar Biol. 2017; 40(6): 1185–1196.

8. Van De Vijver B, Sterken M, Vyverman W, Mataloni G, Nedbalová L, Kopalová K, Elster J, Verleyen E and Sabbe K. Four new non-marine diatom taxa from the Subantarctic and Antarctic regions. Diatom Res. 2010; 25(2): 431-443.

9. Van De Vijver B and Zidarova R. Five new taxa in the genus *Pinnularia* sectio *Distantes* (*Bacillariophyta*) from Livingston Island (South Shetland Islands). Phytotaxa. 2011; 24: 39–50.

10. Van De Vijver B, Zidarova R and De Haan M. Four new *Luticola* taxa (*Bacillariophyta*) from the South Shetland Islands and James Ross Island (Maritime Antarctic Region). Nova Hedwigia. 2011; 92(1): 137–158.

11. Van De Vijver B, Wetzel C, Kopalová K, Zidarova R and Ector L. Analysis of the type material of *Achnanthidium lanceolatum* Brébisson ex Kützing (*Bacillariophyta*) with the description of two new *Planothidium* species from the Antarctic Region. Fottea. 2013; 13(2): 105-117.

12. Van De Vijver B, Kopalová K, Zidarova R and Kociolek JP. Two new *Gomphonema* species (B*acillariophyta*) from the Maritime Antarctic Region. Phytotaxa. 2016; 269(3): 209-220.

13. Van De Vijver B, Lange-Bertalot H, Wetzel CE and Ector L. *Michelcostea*, a new diatom genus (*Bacillariophyta*) from the sub-Antarctic Region. Nova Hedwigia. 2018; 125-136.

14. Zidarova R. Revision of the genus *Hantzschia* (Bacillariophyceae) on Livingston Island (South Shetland Islands, Southern Atlantic Ocean). Plant Ecol~~ogy and~~ Evol~~ution~~. 2010; 143~~ ~~(3): 318–333. https://doi.org/10.5091/plecevo.2010.402

15. Zidarova R, Kopalová K and Van De Vijver B. The genus *Pinnularia* (*Bacillariophyta*) excluding the section *Distantes* on Livingston Island (South Shetland Islands) with the description of twelve new taxa. Phytotaxa. 2012; 44(1):11-37.

16. Zidarova~~,~~ R, Kopalová K, Van de Vijver B~~.~~. ~~et al~~. Diatoms from the Antarctic region~~:~~, ~~maritime~~ Maritime Antarctica. Schmitten-Oberreifenberg: Koeltz Botanical Books, 2016.

17. Guiry MD and Guiry GM. AlgaeBase. 2016. Available at: http://www.algaebase.org (last accessed 16 May 2016).

18. Murdock JN and Dodds WK. Linking benthic algal biomass to stream substratum topography 1. J Phycol. 2007; 43(3): 449-460.

19. Bergey EA. ~~-~~Does rock chemistry affect periphyton accrual in streams?. Hydrobiologia. 2008; 614(1): 141-150.

20. Santos TR and Ferragut C. The successional phases of a periphytic algal community in a shallow tropical reservoir during the dry and rainy seasons. Limnetica. 2013; 32(2): 337-352.

21. Nekola JC and White PS. The distance decay of similarity in biogeography and ecology. J Biogeogr. 1999; 26: 867-878.

22. Wetzel CE, Bicudo DDC, Ector L, Lobo EA, Soininen J, Landeiro VL and Bini LM. Distance decay of similarity in neotropical diatom communities. PLoS One. 2012; 7(9): e45071.

23. Goldenberg Vilar A, Van Dam H, Van Loon EE, Vonk JA, Van Der Geest HG and Admiraal W. Eutrophication decreases distance decay of similarity in diatom communities. Freshw Biol. 2014; 59(7): 1522-1531.

24. Kelly MG, Cazaubon A, Coring E, Dell'Uomo A, Ector L, Goldsmith B, Guasch H, Hurlimann J, Jarlman A, Kawecka B, Kwandrans J, Laugaste R, Lindstrom E, A, Leitao M, Marvan P, Padisaka J, Pipp E, Prygiel J, Rott E, Sabater S, Van Dam H, Vizinet J, ~~-~~Recommendations for the routine sampling of diatoms for water quality assessments in Europe. J Appl Phycol. 1998; 10(2): 215–224.

25. Round FE, Crawford RM and Mann DG. Diatoms: Biology and Morphology of the Genera, Cambridge: Cambridge University Press, 747 p. 1990.

Commented [A30]: I guess you used 'et al' due to the number of authors exceeding a specific number specified by the journal. Otherwise, please consider inserting the entire list
Kelly MG, Cazaubon A, Coring E, Dell'Uomo A, Ector L, Goldsmith B, Guasch H, Hurlimann J, Jarlman A, Kawecka B, Kwandrans J, Laugaste R, Lindstrom E, A, Leitao M, Marvan P, Padisaka J, Pipp E, Prygiel J, Rott E, Sabater S, Van Dam H, Vizinet J,

26. Al-Handal, A.-Y., Riaux-Gobin, C., & and Wulff, A. Cocconeis pottercovei sp. nov. and Cocconeis pinnata var. matsii var. nov., Two new marine diatom taxa from King George Island, Antarctica. Diatom Research., 2010; 25(1), 1-11.

27. Kopalová K, Nedbalova L, De Haan M, Van de Vijver B. Description of five new species of the diatom genus *Luticola* (Bacillariophyta, Diadesmidaceae) found in lakes of James Ross Island (Maritime Antarctic Region). Phyto-taxa. 2011; 27 (1): 44-60. https://doi.org/10.11646/phytotaxa.27.1.5

28. Bonaventura SM, Vinocur A, Allende L, Pizarro H. Algal structure of the littoral epilithon in lentic water bodies at Hope Bay, Antarctic Peninsula. Polar Biology. 2006; 29-(8): 668-680. https://doi.org/10.1007/s00300-005-0104-3

29. Esposito RMM, Spaulding DM, McKnight DM, Van de Vijver B, Kopalová K, Lubinski D, Hall B, Whittaker T. Inland diatoms from the McMurdo Dry Valleys and James Ross Island, Antarctica. Botany. 2008; 86: 1378-1392. https://doi.org/10.1139/B08-100

30. Kawecka B, Olech M, Nowogrodzka-Zagórska M, Wojtuń B. Diatom communities in small water bodies at H. Arctowski Polish Antarctic Station (King George Island, South Shetland Islands, Antarctica). Polar bBiologybiology. 1998; 19-(3): 183-192. https://doi.org/10.1007/s003000050233

31. Lobo EA, Callegaro VLM, Oliveira MA. Diatoms from two lakes of Peninsula Keller, King George Island, Antartic. Revista de Pesquisa Série Botânica. 1998; 10-(1): 3-25.

32. Luo W, Li H, Gao S, Yu Y, Lin L, Zeng Y. Molecular diversity of microbial eukaryotes in sea water from Fildes Peninsula, King George Island, Antarctica. Polar Biology. 2016; 39-(4): 605-616. https://doi.org/10.1007/s00300-015-1815-8

33. Mataloni G, Vinocur A, Pinto PT. Abiotic characterization and epilithic communities of a naturally enriched stream at Cierva Point, Antarctic Peninsula. Antarctic Science. 2005; 17—(2): 163-170. https://doi.org/10.1017/S0954102005002579

34. Pizarro H, Vinocur A. Epilithic biomass in an outflow stream at Potter Peninsula, King George Island, Antarctica. Polar biologyBiol, 2000; 23: 851-857. https://doi.org/10.1007/s003000000164

35. Procopiak LK, Fernandes LF. Valve morphology of the benthic diatom *Fallacia marnieri* (Manguin) Witkowski (Sellaphoraceae-Bacillariophyta). Brazilian Journal of Biology. 2003; 63-(1): 113-119. https://doi.org/10.1590/S1519-69842003000100015

36. Van de Vijver B, Gremmen N, Smith V. Diatom communities from the sub-Antarctic Prince Edward Islands: di-versity and distribution patterns. Polar Biology. 2008; 31-(7): 795-808. https://doi.org/10.1007/s00300-008-0418-z

37. Van de Vijver B, Mataloni G. New and interesting species in the genus *Luticola* DG Mann (Bacillariophyta) from Deception Island (South Shetland Islands). Phycologia. 2008; 47-(5): 451-467. https://doi.org/10.2216/07-67.1

38. Van de Vijver B, Agius JT, Gibson JA, Quesada A. An unusual spine-bearing *Pinnularia* species from the Antarc-tic Livingston Island (South Shetland Islands). Diatom researchRes. 2009; 24—(2): 431-441. https://doi.org/10.1080/0269249X.2009.9705812

39. Van de Vijver B, Beyens L. Freshwater diatom communities of the Strømness Bay area, South Georgia. Antarctic Science. 1996; 8-(4): 359-368. https://doi.org/10.1017/S0954102096000533

40. Van de Vijver B, Kopalová K. Four *Achnanthidium* species (Bacillariophyta) formerly identified as *Achnanthidium minutissimum* from the Antarctic Region. European Journal of Taxonomy. 2014; 79: 1-19. https://doi.org/10.5852/ejt.2014.79

41. Van de Vijver B, Wetzel CE, Ector L. Analysis of the type material of *Planothidium delicatulum* (Bacillariophyta) with the description of two new *Planothidium* species from the sub-Antarctic Region. Fottea. 2018; 18-(2): 200-211. https://doi.org/10.5507/fot.2018.006

42. Vinocur A, Maidana NI. Spatial and temporal variations in moss-inhabiting summer diatom communities from Potter Peninsula (King George Island, Antarctica). Polar Biology.. 2010; 33—(4): 443–455. https://doi.org/10.1007/s00300-009-0719-x

43. Zidarova R, Kopalová K and Van De Vijver B. Ten new *Bacillariophyta* species from James Ross Island and the South Shetland Islands (Maritime Antarctic Region). Phytotaxa. 2016; 272(1): 37-62.

44. Filgueiras BK, Tabarelli M, Leal IR, Vaz-De-Mello FZ, Peres CA and Iannuzzi -L. Spatial replacement of dung beetles in edge-affected habitats: biotic homogenization or divergence in fragmented tropical forest landscapes?. Divers Distrib. 2016; 22(4): 400-409.

45. Colwell RK. EstimateS: Statistical estimation of species richness and shared species from samples. Available at: http://viceroy.eeb.uconn.edu/EstimateS (last accessed 11 June 2017). 2013.

46. Bitencourt BS and da Silva- PG. Forest regeneration affects dung beetle assemblages (*Coleoptera*: *Scarabaeinae*) in the southern Brazilian Atlantic Forest. J Insect Conserv. 2016; 20(5): 855-866.

47. Jost L. Entropy and diversity. Oikos. 2006; 113(2): 363-375.

48. Jost L. Partitioning diversity into independent alpha and beta components. Ecology. 2007; 88(10): 2427-2439.

49. Marcon E and Hérault B. Entropart: An R Package to Measure and Partition Diversity. Available at: http://CRAN.R-project.org/package=entropart (last accessed 11 June 2017). 2014.

50. Manly BF. A method for the estimation of parameters for natural stage-structured populations. Res Popul Ecol. 1997; 39(2): 101-111.

51. Oksanen J, Blanchet FG, Kindt R, Legendre P, O'Hara RB, Simpson GL, Solymos P, Stevens MHH, Wagner H. Vegan: community ecology package. Available at: http://cran.r-project.org/web/packages/vegan/index.html (last accessed 11 June 2017). 2016.

52. Baselga A. Partitioning the turnover and nestedness components of beta diversity. Glob Ecol Biogeogr. 2010; 19(1): 134-143.

Commented [A31]: Perhaps a software is an exception, but please consider to list all authors.

53. Baselga A. Separating the two components of abundance-based dissimilarity: balanced changes in abundance vs. abundance gradients. Methods Ecol Evol. 2013; 4(6): 552-557.

54. Baselga A and Orme -CDL. Betapart: an R package for the study of beta diversity. Methods Ecol Evol. 2012; 3(5): 808-812.

55. Putzke J and Pereira AB. The Antarctic mosses, Canoas, Brazil: Editorial ULBRA, 196 p. 2001.

56. Majewska R, Gambi MC, Totti CM and De Stefano M. Epiphytic diatom communities of Terra Nova Bay, Ross Sea, Antarctica: structural analysis and relations to algal host. AntarcticAntartic Science. 2013; 25(4): 501-513.

57. Trivelpiece, W.Z., Hinke, J.T., Miller, A.K, Reiss, C.S., Trivelpiece, S.G. & and Watter, G.M. Variability in krill biomass links harvesting and climate warming to penguin population changes in Antarctica. P Natl Acad Sci USAProceedings of the National Acaddemy of Sciences. 2011; 108(18), 7625-7628.

58. Schaefer, -C.E.G.R.,- Simas, F.B.NFNB,- Albuquerque-Filho, –M.R., Michel, R.F.M, Viana, J. &and Tatur, H.M. (2004). Fosfatização: processo de formação de solos na Baía- do Almirantado e implicações ambientais. In: En C.E. G.R. Schaefer CEGR, R. Francelino MR, F.N.B. Simas FNB, R. Al–buquerque--Filho MR (Eds.). -Ecossistemas costeiros e monitoramento ambiental da Antártica Marítima: Baía –do Almirantado, Ilha Rei George [Coastal ecosystems and environmental monitoring of Maritime Antarctica; Admiralty Bay, King George Island]-(pp. 47-59). 2004. (pp. 47-59). Viçosa:NEPUT-Departamento de Solos.

59. Shmida AVI and Wilson MV. Biological determinants of species diversity. Journal of Biogeography. 1985;1-20.

60. Sutherland DL. Surface-associated diatoms from marine habitats at Cape Evans, Antarctica, including the first record of living *Eunotogramma marginopunctatum*. Polar Biol. 2008; 31(7): 879-888.

61. Mouquet N and Loreau M. Community patterns in source-sink metacommunities. American Naturalist. 2003; 162(5): 544-557.

62. Maidana NI, Izaguirre I, Vinocur A, Mataloni G and Pizarro H. Diatomeas en una transecta patago□nico-anta□rtica. Ecolog□ıa Austral. 2005; 15: 159-176.

63. Izaguirre I, Saad JF, Schiaffino MR, Vinocur A, Tell G, Sa□nchez ML, Allende L and Sinistro R. Drivers of phy-toplankton diver- sity in Patagonian and Antarctic lakes across a latitudinal gradient (2150 km): the importance of spatial and environ- mental factors. Hydrobiologia. 2016; 764: 157-170.

64. Schiaffino MR, Unrein F, Gasol JM, Massana R, Balague□V and Izaguirre I. Bacterialcommunity struc-ture in a latitudinal gradient of lakes: the roles of spatial versus environmental factors. Freshw Biol. 2011; 56: 1973-1991.

Commented [A32]: English translation is optional.

Formatted: Font: (Default) Times New Roman, Check spelling and grammar

FIGURE LEGENDS

Figure 1. Map of the location of sampling points where *Prasiola crispa* was collected in Antarctica. Ardley Island (62°12'40.40"S 058°55'38.70"W), Halfmonn Island (62º35'41,5"S 059º55'10,1"W) and King George Island: Copacabana (62°10'38,4"S 058°26'57,3"W), Punta Plaza (62°5'14.69"S 058°23'35,21"W), Steinhoose (62°04'18,9"S 058°22'28,2"W) and Voureal (62º10'2,6"S 058º17'52.0"W). The Scar Antarctic Digital Database was used as background shapefile to produce this image and the authors follow the Licence Terms and Conditions under de CC-BY

disclamer. More information can be accessed at https://add.data.bas.ac.uk/repository/entry/show?entryid=f477219b-9121-44d6-afa6-d8552762dc45.

Figure 2 (1-27). Taxa of diatoms in LM. 1. *Achnanthes* sp, 2. *Achnanthidium aff indistinctum*, 3. *Cyclotella meneghiniana*, 4-10. *Cocconeis pinnata* var. *matsii*, 11. *Eunotia aff pseudopaludosa*, 12. *Fragilaria* cf *parva*, 13. *Hantzschia amphioxys*, 14-17. *Luticola amoena*, 18-21. *Luticola austroatlantica*, 22-25. *Luticola muticopsis*, 26. *Luticola olegsakharovii e* 27. *Mayamaea* cf *atomus*. LM scale bar = 10 µm.

Figure 3 (28-60). 28-31. Taxa of diatoms in LM. *Navicula aff perminuta*, 32-34. *Pinnularia australoschoenfelderi*, 35-37. *Pinnularia austroshetlandica*, 38-41. *Pinnularia borealis*, 42-44. *Pinnularia aff microstauron*, 45-48. *Psammothidium germainii*, 49. *Psammothidium papilio*, 50. *Psammonthidium rostrogermainii*, 51-55. *Pseudogomphonema kamtschaticum*, 56-59. *Pteroncola carlinii* e 60. *Thalassiosira gracilis var.expecta*. LM scale bar = 10 µm.

Figure 4 (61-80). 61-63. Taxa of diatoms in SEM. *Cocconeis pinnata* var. *matsii* , 64-65. *Luticola amoena*, 66-68. *Luticola multicopsis*, 69-71. *Navicula aff perminuta*, 72-73. *Pinnularia austroshetlandica*, 74-75. *Psammothidium germainii*, 76-77. *Pseudogomphonema kamtschaticum* e 78-80. *Pteroncola carlinii*.

Figure 5. Rarefaction curves of the diatom species associated with *Prasiola crispa* for the sampled sites. The vertical line represents the point of comparison between the islands. Confidence intervals were omitted for better visualization. Ard = Ardley; Cop = Copacabana; Hal = Half Moon; PuP = Punta Plaza; Ste = Steinhoose; Vou= Voureal.

Figure 6. Plots of abundance (proportional [pi]) of the diatom species associated with *Prasiola crispa* for the sampled sites. The abbreviations refer to the names of the species in Table 2.

Figure 7. Average dissimilarity between sampling points of the diatom species associated with *Prasiola crispa* for the sampled sites based on the presence / absence of species (A) and abundance (B).

Figure 8. Similarity of Jaccard (A) decomposed into components of substitution (B) and nesting (C), and similarity of Bray-Curtis (D) decomposed into components of balanced variation of abundance (E) and abundance gradients (F) of the diatom community associated with *Prasiola crispa* related to the geographical distance between the sampling sites.

Al-Handal, A. Y., Riaux-Gobin, C., & Wulff, A. (2010). Cocconeis pottercovei sp. nov. and Cocconeis pinnata var. matsii var. nov., Two new marine diatom taxa from King George Island, Antarctica. Diatom Research, 25(1), 1-11.

Baselga A. 2010. Partitioning the turnover and nestedness components of beta diversity. Glob Ecol Biogeogr 19(1): 134-143.

Baselga A and Orme CDL. 2012. Betapart: an R package for the study of beta diversity. Methods Ecol Evol 3(5): 808-812.

Baselga A. 2013. Separating the two components of abundance-based dissimilarity: balanced changes in abundance vs. abundance gradients. Methods Ecol Evol 4(6): 552-557.

Bergey EA. 2008. Does rock chemistry affect periphyton accrual in streams?. Hydrobiologia 614(1): 141-150.

Bitencourt BS and da Silva PG. 2016. Forest regeneration affects dung beetle assemblages (*Coleoptera*: *Scarabaeinae*) in the southern Brazilian Atlantic Forest. J Insect Conserv 20(5): 855-866.

Bonaventura SM, Vinocur A, Allende L, Pizarro H (2006) Algal structure of the littoral epilithon in lentic water bodies at Hope Bay, Antarctic Peninsula. Polar Biology 29 (8): 668-680. https://doi.org/10.1007/s00300-005-0104-3

Colwell RK. 2013. EstimateS: Statistical estimation of species richness and shared species from samples. Available at: http://viceroy.eeb.uconn.edu/EstimateS (last accessed 11 June 2017).

Formatted: Space Before: 6 pt

Formatted: Font: Check spelling and grammar

Esposito RMM, Spaulding DM, McKnight DM, Van de Vijver B, Kopalová K, Lubinski D, Hall B, Whittaker T (2008) Inland diatoms from the McMurdo Dry Valleys and James Ross Island, Antarctica. Botany 86: 1378-1392. https://doi.org/10.1139/B08-100

Filgueiras BK, Tabarelli M, Leal IR, Vaz-De-Mello FZ, Peres CA and Iannuzzi L. 2016. Spatial replacement of dung beetles in edge-affected habitats: biotic homogenization or divergence in fragmented tropical forest landscapes?. Divers Distrib 22(4): 400-409.

Goldenberg Vilar A, Van Dam H, Van Loon EE, Vonk JA, Van Der Geest HG and Admiraal W. 2014. Eutrophication decreases distance decay of similarity in diatom communities. Freshw Biol 59(7): 1522-1531.

Guiry MD and Guiry GM. 2016. AlgaeBase. Available at: http://www.algaebase.org (last accessed 16 May 2016).

1. Hamsher S, Kopalova K, Kociolek JP, Zidarova R and Van De Vijver B. 2016. The genus *Nitzschia* on the South Shetland Islands and James Ross Island. Fottea/Czech Phycological Society. Praha, Czech Republic, 2007, currens. 16(1): 79-102.

Heino J, Melo AS and Bini LM. 2015. Reconceptualising the beta diversity-environmental heterogeneity relationship in running water systems. Freshw Biol 60(2): 223-235.

Hubbell SP. 2001. A unified neutral theory of biodiversity and biogeography, Princeton, NJ: Princeton University Press, 392 p.

Jamoneau A, Passy SI, Soininen J, Leboucher T and Tison-Rosebery J. 2018. Beta diversity of diatom species and ecological guilds: Response to environmental and spatial mechanisms along the stream watercourse. Freshw Biol 63(1): 62-73.

Jost L. 2006. Entropy and diversity. Oikos 113(2): 363-375.

Jost L. 2007. Partitioning diversity into independent alpha and beta components. Ecology 88(10): 2427-2439.

Kawecka B, Olech M, Nowogrodzka-Zagórska M, Wojtuń B (1998) Diatom communities in small water bodies at H. Arctowski Polish Antarctic Station (King George Island, South Shetland Islands, Antarctica). Polar biology 19 (3): 183-192. https://doi.org/10.1007/s003000050233

Kelly MG, et al. 1998. Recommendations for the routine sampling of diatoms for water quality assessments in Europe. J Appl Phycol 10(2): 215-224.

Kochman-Kedziora N, Noga T, Zidarova R, Kopalová K and Van DE Vijver B. 2016. *Humidophila komarekiana* sp. nov.(*Bacillariophyta*), a new limnoterrestrial diatom species from King George Island (Maritime Antarctica). Phytotaxa 272(3): 184-190.

Kochman-Kedziora N, Noga T, Van De Vijver B and Stanek-Tarkowska J. 2017. A new *Muelleria* species (*Bacillariophyta*) from the Maritime Antarctic Region. Fottea/Czech Phycological Society. Praha, Czech Republic, 2007, currens. 17(2):264-268.

Kochman-Kedziora N, Pinseel E, Rybak M, Noga T, Olech M And Van De Vijver B. 2018. *Pinnularia subcatenaborealis* sp. nov.(*Bacillariophyta*) a new chain-forming diatom species from King George Island (Maritime Antarctica). Phytotaxa 364(3): 259-266.

Kociolek JP, Kopalová K, Hamsher SE, Kohler TJ, Van De Vijver B, Convey P and Mcknight DM. 2017. Freshwater diatom biogeography and the genus *Luticola*: an extreme case of endemism in Antarctica. Polar Biol 40(6): 1185-1196.

Kopalová K, Nedbalova L, De Haan M, Van de Vijver B (2011) Description of five new species of the diatom genus *Luticola* (Bacillariophyta, Diadesmidaceae) found in lakes of James Ross Island (Maritime Antarctic Region). Phytotaxa 27 (1): 44-60. https://doi.org/10.11646/phytotaxa.27.1.5

Kopalová K, Nedbalová L, Nývlt D, Elster J, Van de Vijver B (2013) Diversity, ecology and biogeography of the freshwater diatom communities from Ulu Peninsula (James Ross Island, NE Antarctic Peninsula). Polar Biology 36 (7): 933-948. https://doi.org/10.1007/s00300-013-1317-5

3. Kopalova K, Ochyra R, Nedbalova L and Van De Vijver B. 2014. Moss-inhabiting diatoms from two contrasting Maritime Antarctic islands. Plant Ecol Evol 147(1): 67–84.

Kopalová K, Kociolek JP, Lowe RL, Zidarova R, Van de Vijver B (2015) Five new species of the genus *Humidophila* (Bacillariophyta) from the Maritime Antarctic Region. Diatom Research 30 (2): 117-131. https://doi.org/10.1080/0269249X.2014.998714

Lobo EA, Callegaro VLM, Oliveira MA (1998) Diatoms from two lakes of Peninsula Keller, King George Island, Antartic. Revista de Pesquisa Série Botânica 10 (1): 3-25.

Luo W, Li H, Gao S, Yu Y, Lin L, Zeng Y (2016) Molecular diversity of microbial eukaryotes in sea water from Fildes Peninsula, King George Island, Antarctica. Polar Biology 39 (4): 605-616. https://doi.org/10.1007/s00300-015-1815-8

Majewska R, Gambi MC, Totti CM and De Stefano M. 2013. Epiphytic diatom communities of Terra Nova Bay, Ross Sea, Antarctica: structural analysis and relations to algal host. Ant Sci 25(4): 501–513.

Manly BF.1997. A method for the estimation of parameters for natural stage-structured populations. Res Popul Ecol 39(2): 101–111.

Marcon E and Hérault B. 2014. Entropart: An R Package to Measure and Partition Diversity. Available at: http://CRAN.R-project.org/package=entropart (last accessed 11 June 2017).

Mataloni G, Vinocur A, Pinto PT (2005) Abiotic characterization and epilithic communities of a naturally enriched stream at Cierva Point, Antarctic Peninsula. Antarctic Science 17 (2): 163-170. https://doi.org/10.1017/S0954102005002579

Mouquet N and Loreau M. 2003. Community patterns in source-sink metacommunities. American Naturalist 162(5): 544-557.

Murdock JN and Dodds WK. 2007. Linking benthic algal biomass to stream substratum topography 1. J Phycol 43(3):449-460.

Nekola JC and White PS. 1999. The distance decay of similarity in biogeography and ecology. J Biogeogr 26: 867-878.

Oksanen J, et al. 2016. Vegan: community ecology package. Available at: http://cran.r-project.org/web/packages/vegan/index.html (last accessed 11 June 2017).

Pizarro H, Vinocur A (2000) Epilithic biomass in an outflow stream at Potter Peninsula, King George Island, Antarctica. Polar biology 23: 851-857. https://doi.org/10.1007/s003000000164

Procopiak LK, Fernandes LF (2003) Valve morphology of the benthic diatom *Fallacia marnieri* (Manguin) Witkowski (Sellaphoraceae-Bacillariophyta). Brazilian Journal of Biology 63 (1): 113-119. https://doi.org/10.1590/S1519-69842003000100015

Putzke J and Pereira AB. 2001.The Antarctic mosses, Canoas, Brazil: Editorial ULBRA, 196 p.

R Core Team. 2017. R: A language and environment for statistical computing. Available at: http://www.r-project.org/ (last accessed 11 June 2017).

Round FE, Crawford RM and Mann DG. 1990. Diatoms: Biology and Morphology of the Genera, Cambridge: Cambridge University Press, 747 p.

Schaefer, C.E.G.R., Simas, F.B.N, Albuquerque-Filho, M.R., Michel, R.F.M, Viana, J. &Tatur, H.M. (2004). Fosfatização: processo de formação de solos na Baía do Almirantado e implicações ambientais. En C.E. G.R. Schaefer, R. Francelino, F.N.B. Simas, R. Al- buquerque Filho (Eds.). Ecossistemas costeiros e monitoramento ambiental da Antártica Marítima: Baía do Almirantado, Ilha Rei George (pp. 47-59). Viçosa:NEPUT-Departamento de Solos.

Shmida AVI and Wilson MV. 1985. Biological determinants of species diversity. J Biogeogr 1-20.

Santos TR and Ferragut C. 2013. The successional phases of a periphytic algal community in a shallow tropical reservoir during the dry and rainy seasons. Llimnetica 32(2): 337-352.

Sutherland DL. 2008. Surface associated diatoms from marine habitats at Cape Evans, Antarctica, including the first record of living *Eunotogramma marginopunctatum*. Polar Biol 31(7):879-888.

Trivelpiece, W.Z., Hinke, J.T., Miller, A.K, Reiss, C.S., Trivelpiece, S.G. & Watter, G.M. 2011. Variability in krill biomass links harvesting and climate warming to penguin population changes in Antarctica. Proceedings of the National Acaddemy of Sciences, 108(18), 7625-7628.

Van de Vijver B, Beyens L (1996) Freshwater diatom communities of the Strømness Bay area, South Georgia. Antarctic Science 8 (4): 359-368. https://doi.org/10.1017/S0954102096000533

2. Van De Vijver B, Gremmen NJ and Beyens L. 2005. The genus *Stauroneis* (*Bacillariophyceae*) in the Antarctic region. J Biogeogr. 32(10): 1791-1798.

Van de Vijver B, Gremmen N, Smith V (2008) Diatom communities from the sub-Antarctic Prince Edward Islands: diversity and distribution patterns. Polar Biology 31 (7): 795-808. https://doi.org/10.1007/s00300-008-0418-z

Van de Vijver B, Mataloni G (2008) New and interesting species in the genus *Luticola* DG Mann (Bacillariophyta) from Deception Island (South Shetland Islands). Phycologia 47 (5): 451-467. https://doi.org/10.2216/07-67.1

Van de Vijver B, Agius JT, Gibson JA, Quesada A (2009) An unusual spine-bearing *Pinnularia* species from the Antarctic Livingston Island (South Shetland Islands). Diatom research 24 (2): 431-441. https://doi.org/10.1080/0269249X.2009.9705812

Van De Vijver B, Sterken M, Vyverman W, Mataloni G, Nedbalová L, Kopalová K, Elster J, Verleyen E and Sabbe K. 2010. Four new non-marine diatom taxa from the Subantarctic and Antarctic regions. Diatom Res 25(2): 431-443.

Van De Vijver B and Zidarova R. 2011a. Five new taxa in the genus *Pinnularia* sectio *Distantes* (*Bacillariophyta*) from Livingston Island (South Shetland Islands). Phytotaxa. 24: 39–50.

Van De Vijver B, Zidarova R and De Haan M. 2011b. Four new *Luticola* taxa (*Bacillariophyta*) from the South Shetland Islands and James Ross Island (Maritime Antarctic Region). Nova Hedwigia 92(1): 137–158.

Van De Vijver B, Wetzel C, Kopalová K, Zidarova R and Ector L. 2013. Analysis of the type material of *Achnanthidium lanceolatum* Brébisson ex Kützing (*Bacillariophyta*) with the description of two new *Planothidium* species from the Antarctic Region. Fottea. 13(2): 105–117.

Van de Vijver B, Kopalová K (2014) Four *Achnanthidium* species (Bacillariophyta) formerly identified as *Achnanthidium minutissimum* from the Antarctic Region. European Journal of Taxonomy 79: 1–19. https://doi.org/10.5852/ejt.2014.79

Van De Vijver B, Kopalová K, Zidarova R and Kociolek JP. 2016. Two new *Gomphonema* species (B*acillariophyta*) from the Maritime Antarctic Region. Phytotaxa. 269(3): 209–220.

Van De Vijver B, Lange-Bertalot H, Wetzel CE and Ector L. 2018. *Michelcostea*, a new diatom genus (*Bacillariophyta*) from the sub-Antarctic Region. Nova Hedwigia 125-136.

Van de Vijver B, Wetzel CE, Ector L (2018) Analysis of the type material of *Planothidium delicatulum* (Bacillariophyta) with the description of two new *Planothidium* species from the sub-Antarctic Region. Fottea 18 (2): 200–211. https://doi.org/10.5507/fot.2018.006

Vinocur A, Izaguirre I (1994) Freshwater algae (excluding Cyanophyceae) from nine lakes and pools of Hope Bay, Antarctic Peninsula. Antarctic Science 6 (4): 483–489. https://doi.org/10.1017/S0954102094000738

Vinocur A, Maidana NI (2010) Spatial and temporal variations in moss-inhabiting summer diatom communities from Potter Peninsula (King George Island, Antarctica). Polar Biology 33 (4): 443–455. https://doi.org/10.1007/s00300-009-0719-x

Wetzel CE, Bicudo DDC, Ector L, Lobo EA, Soininen J, Landeiro VL and Bini LM. 2012. Distance decay of similarity in neotropical diatom communities. PLoS One 7(9): e45071.

Zelazna-Wieczorek J and Olszynski RM. 2016. Taxonomic revision of *Chamaepinnularia krookiformis* Lange-Bertalot et Krammer with a description of *Chamaepinnularia plinskii* sp. nov. Fottea. 16: 112–121.

Zidarova R (2010) Revision of the genus *Hantzschia* (Bacillariophyceae) on Livingston Island (South Shetland Islands, Southern Atlantic Ocean). Plant Ecology and Evolution 143 (3): 318–333. https://doi.org/10.5091/plecevo.2010.402

Zidarova R, Kopalová K and Van De Vijver B. 2012. The genus *Pinnularia* (*Bacillariophyta*) excluding the section *Distantes* on Livingston Island (South Shetland Islands) with the description of twelve new taxa. Phytotaxa. 44(1):11–37.

ZIDAROVA, R. et al. Diatoms from the Antarctic region: maritime Antarctica. Schmitten Oberreifenberg: Koeltz Botanical Books, 2016

Zidarova R, Kopalová K and Van De Vijver B. 2016. Ten new *Bacillariophyta* species from James Ross Island and the South Shetland Islands (Maritime Antarctic Region). Phytotaxa. 272(1):37–62.

POINT-BY-POINIT RESPONSE TO REVIEWERS

REVIEWER'S COMMENTS:

Reviewer #1:

Language is generally clear, though some minor syntax and spelling editing is required throughout; I note that this journal does not offer copy editing, so this will have to be done carefully during revision. Is the citation format in the text correct for the journal? It is unusual to list both numbered references and fully named author lists together.
Response to reviewer: The authors followed the guidelines for manuscript formatting for submission to Plos One Magazine. However, there were some formatting errors that have now been corrected in this review.

Abstract – the description of the role of distance ('isolation by distance') in structuring (or not) diatom community composition could be reworded a bit to make it clearer: at present the sentence structure seems to suggest initially that distance is being considered from each of the six sampling sites individually (for instance along a transect from a central sampling location at each), and rather it later becomes clear that the intention is to look at distance as a factor between the six sampling locations.
Response to reviewer: Its been corrected, we rewritten the description. The modification is found in the marked copy of manuscript.

Introduction – the opening para should make it clear from the outset that the focus is on diatoms; at present the opening sentences appear to make statements applying to the Antarctic flora more generally – if this is the case then they need expanding into a separate general opening para, before subsequently focussing in on diatoms specifically. However, I think the intention is to focus on diatoms from the outset.
Response to reviewer: Ok, the modification is found in the marked copy of manuscript.

Terminology in the para describing the influence of distance – it should be 'community similarity' rather than 'species similarity'.
Response to reviewer: We change this, the modification is found in the marked copy of manuscript.

Within the methods section there is no reference given to how the diatoms obtained were identified? I appreciate the long list of recent species descriptions early in the Introduction, but something specific should be said in this section. Other than these comments, the approaches and analyses used are appropriate.
Response to reviewer: Ok, we add the references used in detail, the modification is found in the marked copy of manuscript.

Taxonomic authorities – I believe that journal format only requires these to be listed once, so there are many instances where repeat listings can be deleted. If acceptable to the journal, I would suggest placing all authorities within Table 2 and deleting them from the text, which will also help make the text more readable.
Response to reviewer: Ok, we change this, the modification is found in the marked copy of manuscript.

Diversity indices – I am not familiar with what 0D, 1D, 2D annotation means?
Response to reviewer: The explanation is at the bottom of page 8 in the following excerpt: "The alpha diversity was approximately 10 species for species richness and this analysis further showed that the true beta diversity of Shannon (1D) was lower than the richness based on species richness (0D) and based on the inverse concentration of Simpson (2D). This shows that differences between sampling sites occurred due to the contrast between the most abundant and the rarest species of each site (Table 4)."

Overall, while the study found differences in various indices between sites, community similarity was high across the entire study area, emphasised by the highest similarity being found between the most distant sites, and there was no overall relationship between separation distance and diversity. I wonder if this might reflect the overall physical scale of the study area, where most of the sampling sites were actually quite close to each other – in other words does this indicate the scale of the study was not large enough to identify such effects, and that 'isolation' was not a significant factor at this scale? Perhaps a larger geographical sampling scale would lead to identification of such a relationship, as predicted by the study's overall hypothesis?
Response to reviewer: Ok, we added this discussion, the modification is found in the marked copy of manuscript.

Figures – the labels on the diatom photo figs are blurred in my pdf?
Response to reviewer: We do not understand this point. Our figures and the pdf generate in the submission process are in highest quality permited to the jounal guidelines.

Table 1 – I think I would suggest presenting this data in km rather than m
Response to reviewer: Ok, we change this, the modification is found in the marked copy of manuscript.

Reviewer #2:

Diatom identification: In the method section of the manuscript, the authors only referenced Round et al. (1990) as the source for the identification of the diatoms they observed in their samples. This is not suitable as this book by Round et al. only deals with taxonomy at the generic level and some of the genera the authors identified where not even described at the time it was published (e.g. Psammothidium, Mayamaea). It would be much better to list all the papers and flora used for identification. In addition, I found particularly surprising that you did not make use of the flora published by Zidarova et al. (2016) specifically dealing with Maritime Antarctica, including the South Shetlands Islands. In particular, the species the authors identified as Luticola aff beyensis and Luticola muticopsis morphotype 1 may be Luticola amoena and Luticola austroatlantica, respectively, as illustrated in the book by Zidarova et al. (2016). Cocconeis aff costata may also correspond to Cocconeis pinnata var. matsii as described by Al-Handal et al. (2010) from King George Island.
Response to reviewer: Ok, we correted this, the modification is found in the marked copy of manuscript.

Spelling of diatom names: I've found numerous mistakes throughout the text of the manuscript, the legend of the figures and in the tables. Please pay attention to that and correct them.
Response to reviewer: Ok, we correted this, the modification is found in the marked copy of manuscript.

Rarefaction analysis: Taxonomic richness assessed through rarefaction in fact consists in an interpolation to lower (and common to all sample) count size and it results in a loss of information (Giesecke et al. 2014). Moreover, samples standardized by size can have different degrees of completeness, depending on the species-abundance distributions of the assemblages that are compared. A better way is to compare samples of equal completeness, not equal size (Chao and Jost 2012). This can be done by using the species accumulation curve to extrapolate the total species richness of the less complete samples (Béguinot 2015). Several parameters can be used to estimate the completeness of the sampling such as the percentages of species represented by one individual (=singleton) and the sample intensity, i.e. abundance divided by richness (Lopez et al. 2012).
Response to reviewer: The authors understand the suggestion, but evaluate that would not modify the results to a significant extent. Regarding the suggestion to use species represented by only one individual in a given sample to access completeness, it cannot be applied to our data, because such species are eliminated from data.

Comparison of the results: the main result is that spatial distance does not appear to drive differentiation in diatom communities in this study. Maybe this result is due to the fact that the distance covered in the study is relatively small (less than 100 km). Could you compare your result with similar studies dealing with the effect of spatial distance on diatom community composition?
Response to reviewer: Ok, we added this discussion, the modification is found in the marked copy of manuscript.

Conclusion: I think the manuscript would really gain if you could broaden a little the scope of your conclusions with a few sentences. For example, could you discuss about any wider implications regarding the potential effect of further climate warming on the resilience of this community. I read that the penguin populations were threatened as their conditions for nesting are changing (change in local weather with unprecedented rain, prematurely snow melt creating puddles of water on the ground, etc…). How would this affect Prasiola and its associated diatom flora?
Response to reviewer: Ok, we added this discussion, the modification is found in the marked copy of manuscript.

Specific comments:
Page 1: In the title, I would use: Distribution of aerophilous diatom communities … to make it clear what kind of samples were investigated.
Page 1: in the address of the authors, correct spelling of Antarctic (only one "t")
Page 2, in abstract: rephrase such as: …such as the type of substrate…
Page 2: correct spelling of Luticola muticopsis (not multicopsis)
Page 2: add dots after initials such as: D.E. Kellogg et al.
Page 2:… in our samples, …
Page 2: …and abundance of their diatom community…
Page 2: keywords: replace "Diatom" by "aerophilous diatoms", delete "Taxonomy" as this paper does not deal with taxonomy
Page 3: rewrite the sentence such as: The larger the distance between sites, the larger are the environmental variations and, therefore, the difference in species composition tends to be also larger. The following sentence, starting by

“Spatial distance is a factor that influences…” is unclear, please re-write it.
Page 4: quantitative analyses: give the number of valves counted to obtain the relative abundance data
Page 4: the statement starting with “sample sufficiency…” is unclear
Page 4: regarding rarefaction, please see above in general comments
Page 5: separate thousands using a comma such as: 10,000
Page 5: use lower case for “also”
Page 5: …(Oksanen et al. 2016) in R.
Page 5: correct spelling of Pseudogomphonema and carlinii
Page 5: Cocconeis pinnata var. matsii is also a marine species (if it’s the correct identification?)
Page 6: correct spelling for muticopsis (several times)
Page 6: just give the authorities once, when a species is first mentioned. There is no need to repeat them every time thereafter.
Page 7: separate thousands using a comma such as: 10,000
Page 9: delete “1.” before “Hamsher”
Page 9: just use Fottea for title of the journal, delete “Czech Phycological Society – Praha, Czech Republic, 2007, currens.”
Page 9: in the reference by Kochman-Kedziora et al.: just use Fottea for title of the journal, delete “Czech Phycological Society – Praha, Czech Republic, 2007, currens.”
Page 10: delete “3.” before “Kopalova”
Page 11: use capital L for journal title: Limnetica
Page 11: delete “2.” before “Van de Vijver”
Response to reviewer: Ok, we corrected the specific comments above.

Fig. 1: add an insert showing the whole of Antarctica to help the reader immediately visualize the location of this region. Some locations on the map and the legend are in Portuguese (e.g Rei George instead of King George; Sistema de Coordenadas, etc…). Please change all to English. Also correct spelling of British. Also give explanation about the units used on sides of the figure.
Response to reviewer: Ok, we correted this, the modification is found in the marked copy of manuscript.

Figs 2 and 3: indicate to what distance the scale bar corresponds (is it 5 microns?), either directly on the figures or in the legend. In the legend: add “LM photographs”; correct spelling of diatom names, use English instead of Portuguese for morphotype
Response to reviewer: Ok, we correted this, the modification is found in the marked copy of manuscript.

Fig. 4: add “SEM photographs”, correct spelling of diatom names.
Response to reviewer: Ok, we correted this, the modification is found in the marked copy of manuscript.

Table 1: I would give the distances in kilometers instead of meters. If you insist in using meters, at least delete the decimals…
Response to reviewer: Ok, we correted this, the modification is found in the marked copy of manuscript.

Table 2: There are many mistakes in the diatom names:
Muticopsis instead of multicopsis
Cyclotella instead of Ciclotella
Fragilaria instead of Fragillaria
Hantzschia instead of Hantzchia
Carlinii instead of carlini
Pseudogomphonema instead of Pseudoghophonema
Olegsakharovii instead of olegsakharoni
Response to reviewer: Ok, we correted this, the modification is found in the marked copy of manuscript.
