# Supplementary material for: Distribution of aerophilous diatom communities associated with terrestrial green macroalgae in the South Shetland Islands, Maritime Antarctica
Source: PLoS One. 2019 Dec 30;14(12):e0226691. doi: 10.1371/journal.pone.0226691 (PMC6936843; doi:10.1371/journal.pone.0226691)
Supplement: Attachment — Submitted filename: POINT-BY-POINIT RESPONSE TO REVIEWERS.docx [file pone.0226691.s001.docx]

POINT-BY-POINIT RESPONSE TO REVIEWERS

REVIEWER'S COMMENTS:

**Reviewer #1:**

Language is generally clear, though some minor syntax and spelling editing is required throughout; I note that this journal does not offer copy editing, so this will have to be done carefully during revision. Is the citation format in the text correct for the journal? It is unusual to list both numbered references and fully named author lists together.

Response to reviewer: The authors followed the guidelines for manuscript formatting for submission to Plos One Magazine. However, there were some formatting errors that have now been corrected in this review.

Abstract – the description of the role of distance (‘isolation by distance’) in structuring (or not) diatom community composition could be reworded a bit to make it clearer: at present the sentence structure seems to suggest initially that distance is being considered from each of the six sampling sites individually (for instance along a transect from a central sampling location at each), and rather it later becomes clear that the intention is to look at distance as a factor between the six sampling locations.

Response to reviewer: Its been corrected, we rewritten the description. The modification is found in the marked copy of manuscript.

Introduction – the opening para should make it clear from the outset that the focus is on diatoms; at present the opening sentences appear to make statements applying to the Antarctic flora more generally – if this is the case then they need expanding into a separate general opening para, before subsequently focussing in on diatoms specifically. However, I think the intention is to focus on diatoms from the outset.

Response to reviewer: Ok, the modification is found in the marked copy of manuscript.

Terminology in the para describing the influence of distance – it should be ‘community similarity’ rather than ‘species similarity’.

Response to reviewer: We change this, the modification is found in the marked copy of manuscript.

Within the methods section there is no reference given to how the diatoms obtained were identified? I appreciate the long list of recent species descriptions early in the Introduction, but something specific should be said in this section. Other than these comments, the approaches and analyses used are appropriate.

Response to reviewer: Ok, we add the references used in detail, the modification is found in the marked copy of manuscript.

Taxonomic authorities – I believe that journal format only requires these to be listed once, so there are many instances where repeat listings can be deleted. If acceptable to the journal, I would suggest placing all authorities within Table 2 and deleting them from the text, which will also help make the text more readable.

Response to reviewer: Ok, we change this, the modification is found in the marked copy of manuscript.

Diversity indices – I am not familiar with what 0D, 1D, 2D annotation means?

Response to reviewer: The explanation is at the bottom of page 8 in the following excerpt: “The alpha diversity was approximately 10 species for species richness and this analysis further showed that the true beta diversity of Shannon (1D) was lower than the richness based on species richness (0D) and based on the inverse concentration of Simpson (2D). This shows that differences between sampling sites occurred due to the contrast between the most abundant and the rarest species of each site (Table 4).”

Overall, while the study found differences in various indices between sites, community similarity was high across the entire study area, emphasised by the highest similarity being found between the most distant sites, and there was no overall relationship between separation distance and diversity. I wonder if this might reflect the overall physical scale of the study area, where most of the sampling sites were actually quite close to each other – in other words does this indicate the scale of the study was not large enough to identify such effects, and that ‘isolation’ was not a significant factor at this scale? Perhaps a larger geographical sampling scale would lead to identification of such a relationship, as predicted by the study’s overall hypothesis?

Response to reviewer: Ok, we added this discussion, the modification is found in the marked copy of manuscript.

Figures – the labels on the diatom photo figs are blurred in my pdf?

Response to reviewer: We do not understand this point. Our figures and the pdf generate in the submission process are in highest quality permited to the jounal guidelines.

Table 1 – I think I would suggest presenting this data in km rather than m

Response to reviewer: Ok, we change this, the modification is found in the marked copy of manuscript.

**Reviewer #2:**

Diatom identification: In the method section of the manuscript, the authors only referenced Round et al. (1990) as the source for the identification of the diatoms they observed in their samples. This is not suitable as this book by Round et al. only deals with taxonomy at the generic level and some of the genera the authors identified where not even described at the time it was published (e.g. Psammothidium, Mayamaea). It would be much better to list all the papers and flora used for identification. In addition, I found particularly surprising that you did not make use of the flora published by Zidarova et al. (2016) specifically dealing with Maritime Antarctica, including the South Shetlands Islands. In particular, the species the authors identified as Luticola aff beyensis and Luticola muticopsis morphotype 1 may be Luticola amoena and Luticola austroatlantica, respectively, as illustrated in the book by Zidarova et al. (2016). Cocconeis aff costata may also correspond to Cocconeis pinnata var. matsii as described by Al-Handal et al. (2010) from King George Island.

Response to reviewer: Ok, we correted this, the modification is found in the marked copy of manuscript.

Spelling of diatom names: I’ve found numerous mistakes throughout the text of the manuscript, the legend of the figures and in the tables. Please pay attention to that and correct them.

Response to reviewer: Ok, we correted this, the modification is found in the marked copy of manuscript.

Rarefaction analysis: Taxonomic richness assessed through rarefaction in fact consists in an interpolation to lower (and common to all sample) count size and it results in a loss of information (Giesecke et al. 2014). Moreover, samples standardized by size can have different degrees of completeness, depending on the species-abundance distributions of the assemblages that are compared. A better way is to compare samples of equal completeness, not equal size (Chao and Jost 2012). This can be done by using the species accumulation curve to extrapolate the total species richness of the less complete samples (Béguinot 2015). Several parameters can be used to estimate the completeness of the sampling such as the percentages of species represented by one individual (=singleton) and the sample intensity, i.e. abundance divided by richness (Lopez et al. 2012).

Response to reviewer: The authors understand the suggestion, but evaluate that would not modify the results to a significant extent. Regarding the suggestion to use species represented by only one individual in a given sample to access completeness, it cannot be applied to our data, because such species are eliminated from data.

Comparison of the results: the main result is that spatial distance does not appear to drive differentiation in diatom communities in this study. Maybe this result is due to the fact that the distance covered in the study is relatively small (less than 100 km). Could you compare your result with similar studies dealing with the effect of spatial distance on diatom community composition?

Response to reviewer: Ok, we added this discussion, the modification is found in the marked copy of manuscript.

Conclusion: I think the manuscript would really gain if you could broaden a little the scope of your conclusions with a few sentences. For example, could you discuss about any wider implications regarding the potential effect of further climate warming on the resilience of this community. I read that the penguin populations were threatened as their conditions for nesting are changing (change in local weather with unprecedented rain, prematurely snow melt creating puddles of water on the ground, etc…). How would this affect Prasiola and its associated diatom flora?

Response to reviewer: Ok, we added this discussion, the modification is found in the marked copy of manuscript.

Specific comments:
Page 1: In the title, I would use: Distribution of aerophilous diatom communities … to make it clear what kind of samples were investigated.
Page 1: in the address of the authors, correct spelling of Antarctic (only one “t”)
Page 2, in abstract: rephrase such as: …such as the type of substrate…
Page 2: correct spelling of Luticola muticopsis (not multicopsis)
Page 2: add dots after initials such as: D.E. Kellogg et al.
Page 2:… in our samples, …
Page 2: …and abundance of their diatom community…
Page 2: keywords: replace “Diatom” by “aerophilous diatoms”, delete “Taxonomy” as this paper does not deal with taxonomy
Page 3: rewrite the sentence such as: The larger the distance between sites, the larger are the environmental variations and, therefore, the difference in species composition tends to be also larger. The following sentence, starting by “Spatial distance is a factor that influences…” is unclear, please re-write it.
Page 4: quantitative analyses: give the number of valves counted to obtain the relative abundance data
Page 4: the statement starting with “sample sufficiency…” is unclear
Page 4: regarding rarefaction, please see above in general comments
Page 5: separate thousands using a comma such as: 10,000
Page 5: use lower case for “also”
Page 5: …(Oksanen et al. 2016) in R.
Page 5: correct spelling of Pseudogomphonema and carlinii
Page 5: Cocconeis pinnata var. matsii is also a marine species (if it’s the correct identification?)
Page 6: correct spelling for muticopsis (several times)
Page 6: just give the authorities once, when a species is first mentioned. There is no need to repeat them every time thereafter.
Page 7: separate thousands using a comma such as: 10,000
Page 9: delete “1.” before “Hamsher”
Page 9: just use Fottea for title of the journal, delete “Czech Phycological Society – Praha, Czech Republic, 2007, currens.”
Page 9: in the reference by Kochman-Kedziora et al.: just use Fottea for title of the journal, delete “Czech Phycological Society – Praha, Czech Republic, 2007, currens.”
Page 10: delete “3.” before “Kopalova”
Page 11: use capital L for journal title: Limnetica
Page 11: delete “2.” before “Van de Vijver”

Response to reviewer: Ok, we corrected the specific comments above.

Fig. 1: add an insert showing the whole of Antarctica to help the reader immediately visualize the location of this region. Some locations on the map and the legend are in Portuguese (e.g Rei George instead of King George; Sistema de Coordenadas, etc…). Please change all to English. Also correct spelling of British. Also give explanation about the units used on sides of the figure.

Response to reviewer: Ok, we correted this, the modification is found in the marked copy of manuscript.

Figs 2 and 3: indicate to what distance the scale bar corresponds (is it 5 microns?), either directly on the figures or in the legend. In the legend: add “LM photographs”; correct spelling of diatom names, use English instead of Portuguese for morphotype

Response to reviewer: Ok, we correted this, the modification is found in the marked copy of manuscript.

Fig. 4: add “SEM photographs”, correct spelling of diatom names.

Response to reviewer: Ok, we correted this, the modification is found in the marked copy of manuscript.

Table 1: I would give the distances in kilometers instead of meters. If you insist in using meters, at least delete the decimals…

Response to reviewer: Ok, we correted this, the modification is found in the marked copy of manuscript.

Table 2: There are many mistakes in the diatom names:
Muticopsis instead of multicopsis
Cyclotella instead of Ciclotella
Fragilaria instead of Fragillaria
Hantzschia instead of Hantzchia
Carlinii instead of carlini
Pseudogomphonema instead of Pseudoghophonema
Olegsakharovii instead of olegsakharoni
Response to reviewer: Ok, we correted this, the modification is found in the marked copy of manuscript.
