# Supplementary material for: Distribution of aerophilous diatom communities associated with terrestrial green macroalgae in the South Shetland Islands, Maritime Antarctica
Source: PLoS One. 2019 Dec 30;14(12):e0226691. doi: 10.1371/journal.pone.0226691 (PMC6936843; doi:10.1371/journal.pone.0226691)
Supplement: Attachment — Submitted filename: Response to Reviewers.docx [file pone.0226691.s003.docx]

Dear Editor and Reviewers,

we would like to inform you that image 4 of the manuscript "Distribution of aerophilous diatom communities associated with terrestrial green macroalgae in the South Shetland Islands, Maritime Antarctica", with manuscript number PONE-D-19-16073R1 submitted to the journal Plos One, has not been authorized for disclosure or publication, representing a conflict of interest between authors. We have therefore removed it from the updated version of the article. Nevertheless we would like to emphasize that the lack of this image does not change the context of the paper, nor does it, in any way, impair the results described, as the species previously shown in image 4 are also represented in Figures 2 and 3 as follows:

1. *Cocconeis pinnata* var. *matsii* – Cited in Figure 2 (4-10)

2. *Luticola amoena* - Cited in Figure 2 (14-17)

3. *Luticola multicopsis* - Cited in Figure 2 (22-25)

4. *Navicula aff perminuta* - Cited in Figure 3 (28-31)

5. *Pinnularia austroshetlandica* - Cited in Figure 3 (32-34)

6. *Psammothidium germainii* - Cited in Figure 3 (45-48)

7. *Pseudogomphonema kamtschaticum* - Cited in Figure 3 (51-55)

8. *Pteroncola carlinii* - Cited in Figure 3 (56-59)

In response to questions:

Editor Comments:

“1. Please consider more about the roles of rare species in the estimation of species richness. There are many methods in ecology to deal with rare species, rather than simply deleting them from the original data.”

Regarding the suggestion to use species represented by only one individual in a given sample to access completeness, we argue that in such cases we might be examining the result of a contaminant. Species represented by only one valve in each sample were excluded from the analyses due to the difficulty of a correct species determination and to avoid potential contaminants. We have added this fact to the manuscript methodology along with references.

“2. Please consider the comments about distance.”

We added a discussion about it in the manuscript.

“3. Please carefully check the text to kill the typos, some of which have been marked in the attached file from the reviewer.”

Revised text.

Reviewer #2:

“There are however two issues with the reply the authors gave to my comments:

1. “Regarding the suggestion to use species represented by only one individual in a given sample to access completeness, it cannot be applied to our data, because such species are eliminated from data.“ I really don't understand this reply. Species richness (i.e. the number of species present in the sample) is more or less the basis of this study, so why would you "eliminate" the rare species from the data? Please explain? In any case there was no mention of this “elimination” in the method section of the paper.”

Regarding the suggestion to use species represented by only one individual in a given sample to access completeness, we argue that in such cases we might be examining the result of a contaminant. Species represented by only one valve in each sample were excluded from the analyses due to the difficulty of a correct species determination and to avoid potential contaminants. We have added this fact to the manuscript methodology along with references.

“2. I also asked the authors to consider in their discussion that the relatively small distance (less than 100 km) covered in this study may in part explains the lack of relationship between spatial distance and the diatom communities similarity. Note that the other reviewer made the same remark. The authors did not answer this question directly and I could not found any mention of it in the revised version of the manuscript.”

We added a discussion about it in the manuscript.

“In addition, there are still a few typos in the manuscript. Please see the PDF with my comments as “sticky notes”.”

Revised text.
